# Untargeted Metabolomic Characterization of Glioblastoma Intra-Tumor Heterogeneity Using OrbiSIMS

**DOI:** 10.1021/acs.analchem.2c05807

**Published:** 2023-03-30

**Authors:** Wenshi He, Max K. Edney, Simon M. L. Paine, Rian L. Griffiths, David J. Scurr, Ruman Rahman, Dong-Hyun Kim

**Affiliations:** † Centre for Analytical Bioscience, Advanced Materials & Healthcare Technologies Division, School of Pharmacy, University of Nottingham, Nottingham NG7 2RD, U.K.; ‡ Department of Chemical and Environmental Engineering, Faculty of Engineering, University of Nottingham, Nottingham NG7 7RD, U.K.; § Neuropathology Laboratory, Nottingham University Hospitals NHS Trust, Nottingham NG7 2UH, U.K.; ∥ Children’s Brain Tumour Research Centre, Biodiscovery Institute, School of Medicine, University of Nottingham, Nottingham NG7 2RD, U.K.

## Abstract

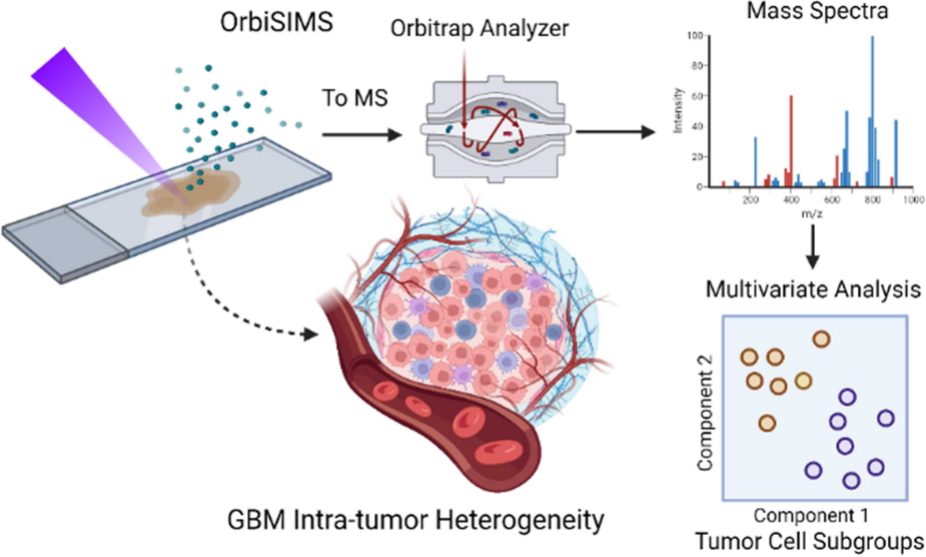

Glioblastoma (GBM) is an incurable brain cancer with a median survival of less than two years from diagnosis. The standard treatment of GBM is multimodality therapy comprising surgical resection, radiation, and chemotherapy. However, prognosis remains poor, and there is an urgent need for effective anticancer drugs. Since different regions of a single GBM contain multiple cancer subpopulations (“intra-tumor heterogeneity”), this likely accounts for therapy failure as certain cancer cells can escape from immune surveillance and therapeutic threats. Here, we present metabolomic data generated using the Orbitrap secondary ion mass spectrometry (OrbiSIMS) technique to investigate brain tumor metabolism within its highly heterogeneous tumor microenvironment. Our results demonstrate that an OrbiSIMS-based untargeted metabolomics method was able to discriminate morphologically distinct regions (viable, necrotic, and non-cancerous) within single tumors from formalin-fixed paraffin-embedded tissue archives. Specifically, cancer cells from necrotic regions were separated from viable GBM cells based on a set of metabolites including cytosine, phosphate, purine, xanthine, and 8-hydroxy-7-methylguanine. Moreover, we mapped ubiquitous metabolites across necrotic and viable regions into metabolic pathways, which allowed for the discovery of tryptophan metabolism that was likely essential for GBM cellular survival. In summary, this study first demonstrated the capability of OrbiSIMS for in situ investigation of GBM intra-tumor heterogeneity, and the acquired information can potentially help improve our understanding of cancer metabolism and develop new therapies that can effectively target multiple subpopulations within a tumor.

## Introduction

Isocitrate dehydrogenase wild-type (IDH WT) glioblastoma (GBM) is the most common and aggressive malignant brain tumor in adults with an invariable global incidence rate of 2.9–10.4 cases per 100,000 person-years.^1−3^ Current standard of care includes maximum safe surgical removal, followed by a standardized regimen of temozolomide chemotherapy and radiotherapy.^4,5^ Despite multimodal treatment, the overall median survival of GBM patients is less than 24 months from first diagnosis and less than 12 months from recurrence.^6−8^ An important reason for treatment failure is the persistence of residual disease cells that are treatment resistant and can inevitably initiate tumor recurrence, which has limited treatment options.^6,9^

Accumulating evidence indicates that the degree of intra-tumor heterogeneity is a key contributor to tumor recurrence and treatment failure.^10−12^ The Cancer Genome Atlas is a landmark cancer genomics program, which molecularly characterized cancers. It has offered important insights into genomic changes and inter-tumoral heterogeneity in a large GBM cohort with identification of molecular subgroups, namely, classical, neural, pro-neural, and mesenchymal.^13,14^ However, it appears to be insufficient to capture the fast-evolving landscape of GBM,^15^ and little therapeutic benefit was gained from subgroup-specific molecular targeted therapeutic designs with only a slight survival advantage of aggressive chemoradiotherapy for the pro-neural subgroup.^14^

More recently, new evidence indicated that intra-tumor heterogeneity may play an even more crucial role.^14,16,17^ Cancer stem cells (CSCs) in GBM are at the apex of an entropic hierarchy and impart devastating therapy resistance.^18^ They demonstrate two principal features, differentiation and self-renewal,^19^ and participate in tissue development and repair in tumors.^20^ CSCs can thrive in harsh, complex microenvironmental niches such as hypoxia, the invasive edge, and the perivascular niche.^21^ These niches do not just harbor GSCs but rather they ensure the growth, maintenance, and protection of cancer cells from immune surveillance and therapeutic threats by exhibiting communication centers within the tumor.

Understanding cancer cellular activities as related to its microenvironment is essential for elucidating the complex landscape of the GBM ecosystem. To do so, an untargeted metabolomics approach can be used to probe biochemical differences between cancer cell subpopulations that reside in different niches within the tumor.^22^ It allows for the measurement of the broadest range of metabolites present in a biological system as an integrated result of both gene expression and its environmental influences.^23^ Although liquid chromatography–mass spectrometry (LC–MS) is a powerful tool frequently applied in untargeted metabolomics, it requires complex and extensive sample preparation procedures and has limited compatibility with formalin-fixed paraffin-embedded (FFPE) tissue archives. In recent years, MS-based surface analysis techniques have provided many new and exciting opportunities in brain tumor research as they enable us to detect molecules directly from the tissue with minimal sample preparation.^24,25^ However, due to the lack of suitable analytical techniques, only few studies describe metabolic intra-tumor heterogeneity in GBM. Recently, Gularyan et al. employed a time-of-flight secondary ion mass spectrometry (ToF-SIMS) method to differentiate morphologically distinct regions within a GBM based on the detected secondary ions.^26^ However, detailed information of altered metabolism was not provided due to excessive fragmentation of analytes during ionization and limited mass resolving power of the ToF analyzer of SIMS instruments (*m*/Δ*m* ∼ 10,000),^27^ which makes it impossible to precisely identify the original molecule that gave rise to the corresponding secondary ion.

A solution to these inherent drawbacks of ToF-SIMS is the recently developed Orbitrap secondary ion mass spectrometry (OrbiSIMS) technique.^28^ This instrument combines traditional ToF-SIMS with Orbitrap MS. The Orbitrap analyzer increases the instrument’s mass resolving power to 240,000 at *m*/*z* 200, which significantly improves the confidence in ion annotation with accurate masses. The technique also employs an argon gas cluster ion beam (GCIB), which is capable of liberating whole molecules from samples due to reduced analyte fragmentation compared with liquid metal ion guns (LMIG) traditionally used in ToF-SIMS, making it particularly applicable to biological materials.^28^ OrbiSIMS has been successfully applied in a range of biological samples, such as undigested proteins from human skin,^29^ metabolites from single cells,^30^ amino acids and lipids from skin,^31^ and small molecules on biofilms.^32^ A previous study conducted by our group combined OrbiSIMS and liquid extraction surface analysis-tandem MS (LESA-MS/MS) to reveal predictive metabolite signatures of pediatric brain tumor relapse using tumor tissue microarrays,^33^ which opened many opportunities to perform in situ metabolomic analysis that may largely improve our understanding of cancer metabolism, an emerging hallmark of cancer.^34^ However, this technique was not previously employed to study metabolic alterations associated with the tumor microenvironment or histopathological features within single tumors.

Here, we performed an untargeted metabolomics analysis directly on human GBM whole tissue sections using OrbiSIMS to identify metabolite signatures associated with distinct histological features in the tumor microenvironment and to discover candidate essential metabolic pathways for functionally disparate GBM cell subpopulations. The same samples analyzed by OrbiSIMS were subjected to subsequent fragmentation and structural characterization with LESA-MS/MS^35^ for accurate identification and confirmation of putatively annotated metabolites that were found to be important in OrbiSIMS analysis. This novel analytical approach could lead to the discovery of personalized treatments that effectively target multiple subgroups of cancer cells predicated on the GBM metabolome.

## Experimental Section

### Sample Preparation

FFPE tissue archives stored for five to seven years from a total of five IDH WT GBM patients were used in this study (SI Table S1). Hematoxylin and eosin (H&E)-stained sections were examined by a neuropathologist at Nottingham University Hospital. This project was approved by the National Research Ethics Committee (NRES Committee East Midlands), and the granted Ethics Reference Number was 11/EM/0076. Histologically heterogeneous tumors that contained necrotic, viable, and non-cancerous (normal brain) regions were chosen for this study. 4 μm sections were cut from the tumor block. The FFPE sections were deparaffinized prior to the OrbiSIMS analysis using a protocol adapted from Meurs et al.^36^ Each section was washed in xylene for 1 min and repeated once in fresh xylene. The deparaffinized tissue section was left for drying in a fume hood for at least an hour prior to the experiments.

### OrbiSIMS Depth Profile Analysis

OrbiSIMS was performed on a Hybrid SIMS instrument (IONTOF, GmbH) with a dual analyzer (ToF and Orbitrap) and a multiple primary and sputter ion beam capability (e.g., Ar GCIB and Bi LMIG) system, as outlined by Passarelli et al.^28^ Prior to analyses, the Orbitrap analyzer was calibrated with silver cluster ions using Bi LMIG.^28^ The experiment was conducted in the low collisional cooling regime. The helium (He) collision cell pressure was set to 0.05 mbar.^37^ To acquire depth profiles from regions of interest (ROIs), a 20 keV Ar_3000_^+^ GCIB with a diameter of 20 μm was operated in quasi-continuous analysis mode with a cycle time of 200 μs on the Q Exactive HF Orbitrap MS. The duty cycle of the beam was set to 4.4%, and the primary current was 262 pA. The mass resolution was set to 240,000 at *m*/*z* 200. The maximum injection time was 500 ms. Depth profiles were collected in negative ion mode at a mass range of *m*/*z* 75–1125 from an area of 200 × 200 μm using a sawtooth raster mode with a crater size of 284.5 × 284.5 μm. The optimal target potential was +56.4 V. For all samples, profiles were taken from characteristic histological regions (necrotic, viable, and non-cancerous) without overlapping craters. Each profile was a sum of 80 scans. Charge compensation was achieved by flooding the sample with a low-energy electron flood gun (21 V) and regulation of the main chamber with argon gas (9 × 10^–7^ mbar).

### OrbiSIMS Chemical Imaging

For the acquisition of GCIB OrbiSIMS images, a 20 keV Ar_3000+_ GCIB analysis beam, focused to 2 μm, was used. The images were collected in negative polarity at a mass range of *m*/*z* 75–1125 with the final pixel size of 3 μm in random raster mode. The cycle time was 200 μs. The duty cycle of the beam was set to 27.78%, and the primary current was 232 pA. The optimal target potential was set to +16.0 V. The mass resolution was set to 240,000 (at *m*/*z* 200), and a fixed injection time of 500 ms was used. In the initial optimization experiment, a 20 μm GCIB was used to reduce the image acquisition time. The experiment was conducted in the low collisional cooling regime. The helium (He) collision cell pressure was set to 0.05 mbar. For the acquisition of the LMIG ToF-SIMS image, a 30 keV Bi_3+_ LMIG was operated in negative mode. A total of 512 × 512 pixels were acquired for each 400 μm × 400 μm area (781.25 nm/pixel). Three shots were accumulated for each pixel. Measurements shown were a sum of five scans. Charge compensation was achieved by flooding the sample with a low-energy electron flood gun (21 V) and regulation of the main chamber with argon gas (9 × 10^–7^ mbar) in all imaging experiments.

### OrbiSIMS Data Analysis

Depth profile spectra were exported as a .TXT file from IONTOF SurfaceLab 7 software. Secondary ions of interest were selected automatically (threshold: 0.1% of the base peak intensity) and aligned within a 5 ppm *m*/*z* window using an in-house MATLAB (R2020a, The MathWorks, Inc., Natick, MA) script.^36^ Features with more than 20% missing values were removed, and *k*-nearest neighbor (*k*nn) imputation was used for zero filling for the remaining missing values.^38^ Before subjecting the processed data to statistical analysis, the background peaks were removed. Peak intensities were normalized to the total ion count (TIC) and Pareto scaled.^39^ Principal component analysis (PCA) and orthogonal partial least-squares discriminant analysis (OPLS-DA) models were constructed for class differentiation and feature selection using SIMCA P 16 software (Umetrics, Sweden). OPLS-DA models were validated by the built-in permutation tests. Discriminative ions were discovered by using a variable’s importance in projection (VIP) score ≥1 in OPLS-DA and *p*-value <0.05 in multiple *t*-tests. False discovery rate (FDR) correction on the *p*-value was conducted using the Benjamini–Hochberg procedure (*q* < 0.05).^40^

Orbitrap and ToF images were processed in IONTOF SurfaceLab 7. Small hydrocarbon fragments (C_2_^–^, C_3_^–^, C_4_^–^, and PO_3_^–^) were used for internal mass calibration for data acquired from the ToF analyzer. PCA of the ToF-SIMS image was carried out using a multivariate analysis platform simsMVA.^41^

### Metabolite Identification and Pathway Analysis

Detected peaks were subjected to metabolite identification using the Human Metabolome Database (HMDB) (https://hmdb.ca/)^42^ with a mass tolerance of 5 ppm for level 3 identification by matching the accurate masses. [M–H]– and [M–H–H_2_O]– were included as ion adduct types. Level 2 identification was carried out by matching both the accurate masses and LESA-MS/MS spectra (protocol in Supporting Information, SI) with the experimental reference at the same normalized collision energy in mzCloud (two orthogonal data).^43^ Monoisotopic masses of annotated metabolites were used for pathway mapping by searching against the *Homo sapiens* (Strain: global) (Source: Publication, Version: 2.02) database (2 ppm mass tolerance) in MetExplore.^44^

## Results and Discussion

### Metabolic Characterization of GBM with OrbiSIMS

**Figure 1:**
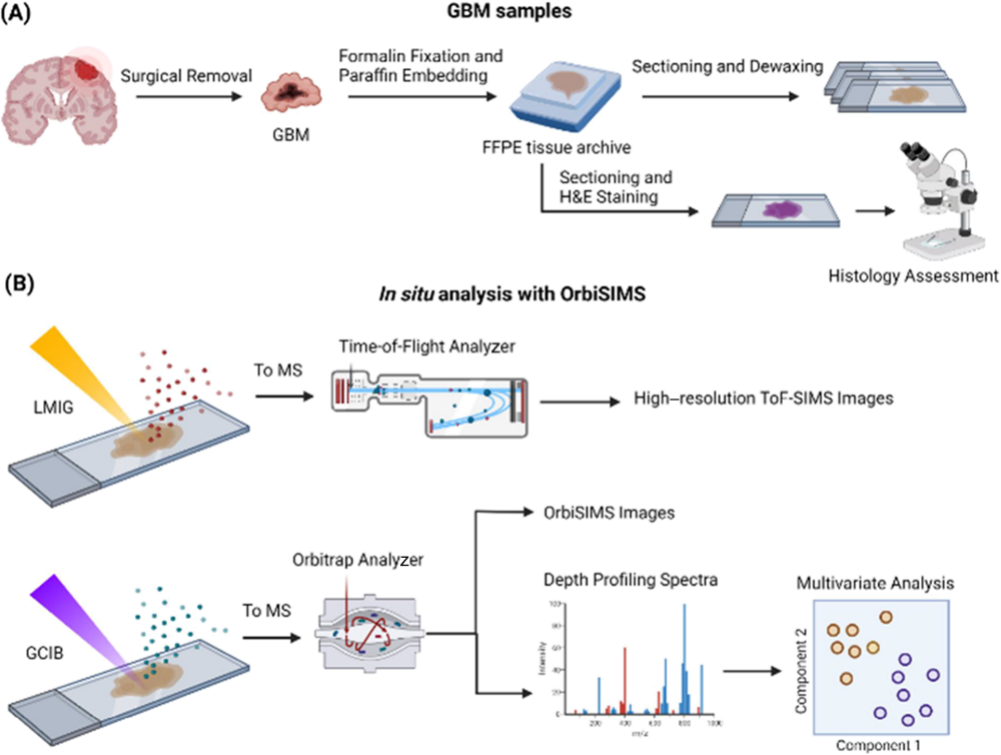
Schematic of workflow. (A) GBM surgical biopsies are fixed with formalin and embedded in paraffin for long-term storage. A tissue section is H&E stained for pathological assessment. The samples for MS analyses are sectioned and dewaxed. (B) Imaging experiments are conducted using Bi LMIG ToF MS and Ar GCIB Orbitrap MS. Representative spectra from histological ROIs are acquired by single-site depth profiling using GCIB Orbitrap, and the ion intensity matrices are subjected to multivariate analysis.

FFPE tumor tissue sections were primarily used in this study (Figure 1A). As part of the initial method development, a tissue section was analyzed in imaging mode using both Bi LMIG coupled to a ToF mass analyzer and Ar GCIB coupled to an Orbitrap analyzer (Figure 1B).^28^

The ToF-SIMS analysis allowed for the rapid identification of potential ROIs where high molecular heterogeneity and rich chemical information were observed on a GBM tissue section (SI Figure S1). Assessing the histology of the tissue by H&E staining, the ROIs contained distinct morphological features in the GBM microenvironment (SI Figure S2A). A GCIB Orbitrap analysis was applied to image the same area for comparison. Higher lateral resolution (approximately 1 μm) and higher speed of analysis (less than 3 min for a 1 mm^2^ area) were achieved with LMIG ToF compared to GCIB Orbitrap (20 μm lateral resolution and >300 min for a 1 mm^2^ area) at its highest mass resolution setting (240,000 at *m*/*z* 200) (SI Figure S2A). However, a drawback of LMIG ToF-SIMS is excessive molecule fragmentation and low mass resolution,^26^ which hinders the identification of important metabolites and further clinical interpretation. Employing GCIB OrbiSIMS, a significant reduction in fragmentation was observed, and many lipid species at *m*/*z* 300–600 were detected (SI Figure S2B).

A total of 168 out of 210 negative ions were putatively annotated in the HMDB using the accurate ion mass owing to the high mass accuracy offered by the Orbitrap analyzer. The most abundant chemical classes were carboxylic acids, benzene and its substitutes, and pyridines and their derivatives (SI Figure S2C). Therefore, the GCIB Orbitrap method was used subsequently in the latter experiments. However, the major limitation with FFPE samples is that certain metabolites and lipids that are found in fresh tissue may not be present in the FFPE tissue. It could be due to the cross-linking in FFPE and loss of analytes during long wash and incubation in organic solvents during formalin fixation and dewaxing prior to analysis.^45^

### Metabolite Profiles Differentiate Viable, Necrotic, and Non-Cancerous Cells

GBM samples containing viable, necrotic, and non-cancerous cells within single tumor samples were analyzed using a GCIB Orbitrap depth profiling method (SI Figure S5A). The metabolite profiles were obtained from multiple sites across each histopathological region (i.e., necrosis, viable tumor cells, and non-cancerous regions) (*N* = 4, *n* = 5) by depth profiling (SI Figure S3). The detailed histology of each analyzed region and the demographic information for the patients are shown in the Supporting Information (SI Figure S4 and Table S1).

**Figure 2:**
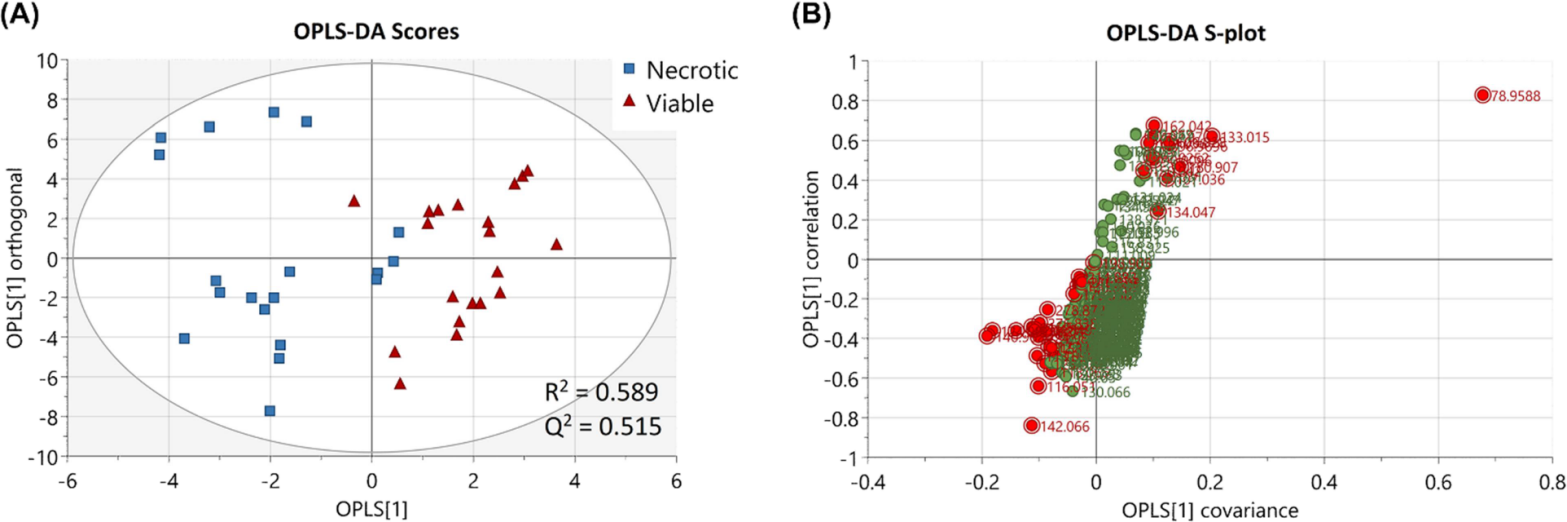
Supervised model with OPLS-DA. (A) Score plot shows clustering of metabolite profiles based on the histological regions (necrotic and viable cells). (B) Important discriminative ions from the OPLS-DA model were revealed by the *S*-plot.

First, a PCA model (*R*^2^ = 0.91, *Q*^2^ = 0.787) was constructed (SI Figure S5). It showed that viable tumor cells clustered distinctly from non-cancerous cells (indicative of predominance of distinct metabolic pathways active within each cell type). The corresponding loading plot revealed metabolites important for separating the groups, including phosphate (*m*/*z* 78.9588), adenine (*m*/*z* 134.047), xanthine (*m*/*z* 133.0154), and indole (*m*/*z* 116.051). Furthermore, a supervised multivariate analysis model was constructed for orthogonal projection to latent structures discriminant analysis (OPLS-DA) to deconvolute the data (Figure 2A). The OPLS-DA model was able to separate necrotic and viable tumor cells (*R*^2^ = 0.589, *Q*^2^ = 0.515), which was validated using leave-one-out cross-validation. A *Q*^2^ value > 0.4 for biological samples is considered to be acceptable for a valid classification mode,^46^ and *Q*^2^ > 0.5 is admitted for good predictability.^47^ The associated *S*-plot showed the covariance and correlation between the ions and the modeled class designation in a scatter plot,^48^ which identified statistically significant and potentially biochemically important metabolites. Ions with a VIP score ≥ 1 were considered discriminative, which were highlighted in the *S*-plot (Figure 2B). OPLS-DA models also revealed differences in metabolite profiles between necrotic and non-cancerous regions (*R*^2^ = 0.571, *Q*^2^ = 0.531) (SI Figure S6A), as well as viable and non-cancerous regions (*R*^2^ = 0.542, *Q*^2^ = 0.734) (SI Figure S6B). To mitigate the issue of potential overfitting, we further validated all OPLS-DA models through a permutation test, which compares the distribution of *Q*^2^ values from the original data to the *Q*^2^ values when original *Y* values are randomly assigned (SI Figure S6C).

**Figure 3:**
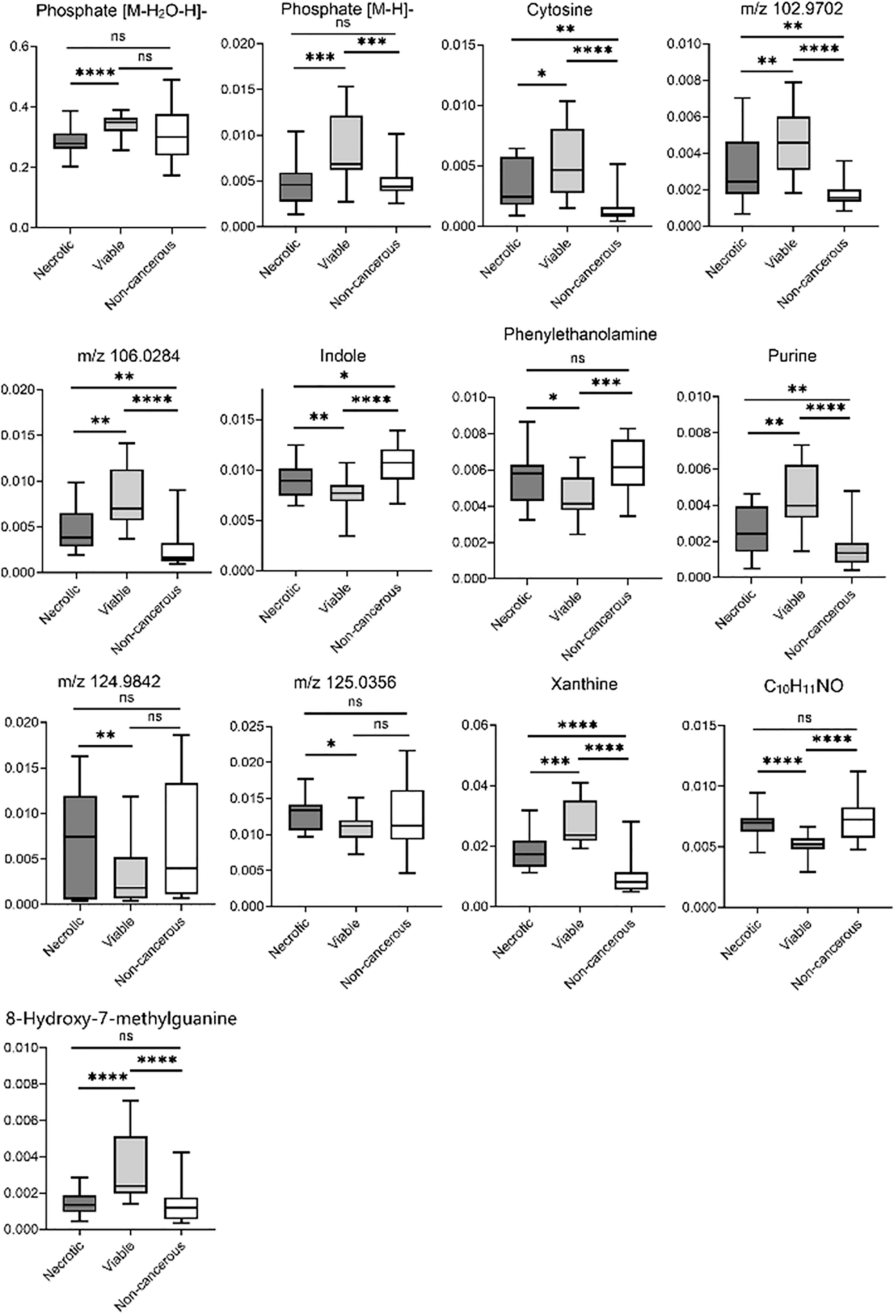
Ion intensities (normalized to the TIC) of discriminative ions between necrotic and viable regions (VIP score ≥ 1 in the OPLS-DA model; *p* < 0.05 using Student’s *t*-test and FDR correction) detected using the single-site depth profiling method (*, *p* < 0.05; **, *p* < 0.01; ***, *p* < 0.001; ****, *p* < 0.0001; ns, not significant).

The discriminative ions discovered in the OPLS-DA model were subjected to Student’s *t*-test to determine how they were altered between viable and necrotic groups. In total, we found 13 ions with significantly different relative abundance (Figure 3), and 9 ions were putatively annotated using the HMDB. The elevated metabolites in viable cancer cells were cytosine (*m*/*z* 92.0252, [M–H_2_O–H]−), phosphate (*m*/*z* 78.9588, [M–H_2_O–H]– and *m*/*z* 96.9696, [M–H]−), purine (*m*/*z* 119.0362, [M–H]−), xanthine (*m*/*z* 133.0154, [M–H_2_O–H]−), and 8-hydroxy-7-methylguanine (*m*/*z* 162.0419, [M–H_2_O–H]−). In GBM, it has been reported that purines can promote DNA repair, therefore correlating with radiotherapy resistance.^49^ It is also an important limiting factor for the growth, proliferation, and maintenance of brain tumor-initiating cells responsible for tumorigenesis.^50^ Another significantly elevated metabolite in viable tumor cells is xanthine, which is one of the downstream metabolites in purine catabolism. It suggests upregulation of purine metabolism in viable GBM cells.^51^ Interestingly, 8-hydroxy-7-methylguanine, a methylated purine compound, was also found to be richer in viable tumor cells compared to necrotic and non-cancerous regions. This is in agreement with previous studies that methylated nucleosides are present in higher amounts in cancer patients compared to healthy individuals.^52,53^ In contrast, indole (*m*/*z* 116.0506, [M–H]−) and phenylethanolamine (*m*/*z* 118.0662, [M–H_2_O–H]−) were found to be less abundant in viable cells compared to the non-cancerous and necrotic regions.

**Figure 4:**
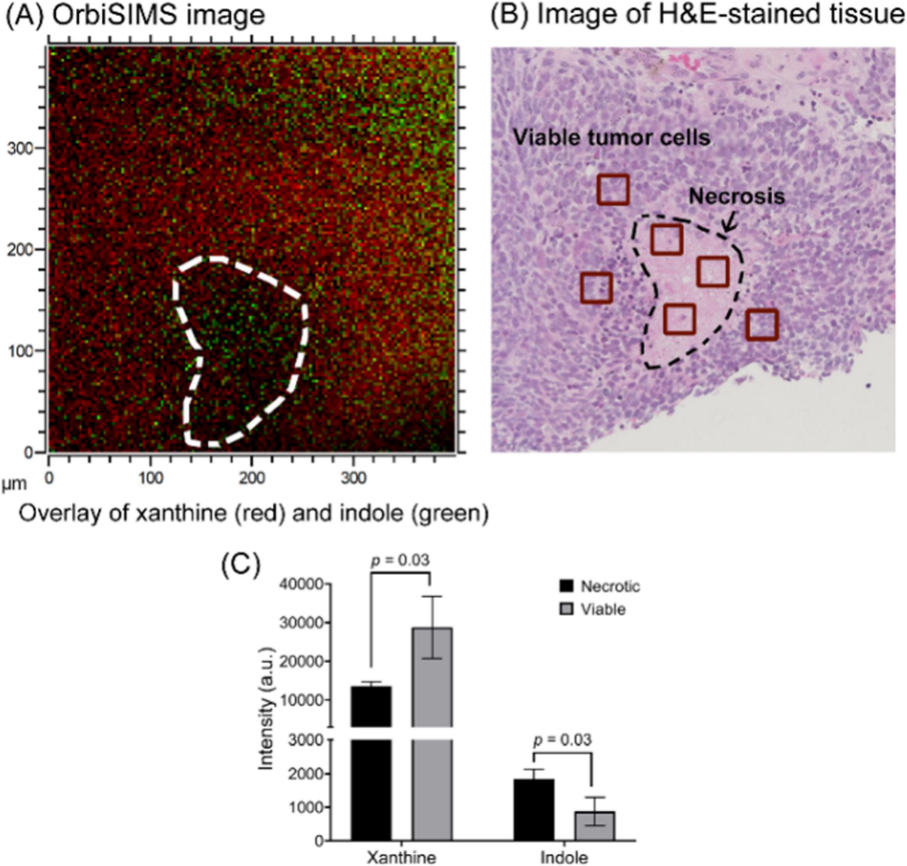
OrbiSIMS imaging. (A) Overlay of xanthine (*m*/*z* 133.0154, red) and indole (*m*/*z* 116.0506, green). The white dashed curve represents the necrotic region. (B) Microscopic image of the H&E-stained tissue. (C) Ion intensities within the representative ROIs (30 × 30 μm) (red squares in B).

As part of the result validation, we further examined the spatial distribution of the distinguishing metabolites (Figure 3) using GCIB OrbiSIMS imaging with high spatial resolution (3 μm) (SI Figure S7). The region was characterized by necrosis surrounded by viable cells (Figure 4B). In line with the depth profiling result, indole (*m*/*z* 116.0506) specifically localizes to the necrotic region, whereas xanthine (*m*/*z* 133.0154) is in a complementary pattern (Figure 4A). The ion intensities of the pixels in the chosen ROIs (red squares in Figure 4B) showed enhancement of indole in the necrotic region (Figure 4C). However, it was more challenging to co-register the ion maps with the histopathological features for some metabolites due to their relatively low ion intensities. This could potentially be improved by increasing the number of scans or using larger ion beams to increase sensitivity for imaging analyses.

### Ubiquitous Metabolic Pathways across Necrotic and Viable Regions

Necrosis is a hallmark feature of GBM,^21^ a powerful predictor of poor prognosis,^54^ and a major factor for treatment resistance.^55^ Identifying ubiquitous pathways that are affected in both necrotic regions and viable cancer cells can potentially open more opportunities for effective treatments. The monoisotopic masses of metabolites that displayed equal abundance across the necrotic and viable regions (VIP score ≥ 1 and *p* > 0.05) were submitted to MetExplore^44^ for metabolic pathway analysis, which allowed for putative identification of ubiquitous metabolic pathways across these two histologically and functionally distinct regions (SI Table S2).

**Figure 5:**
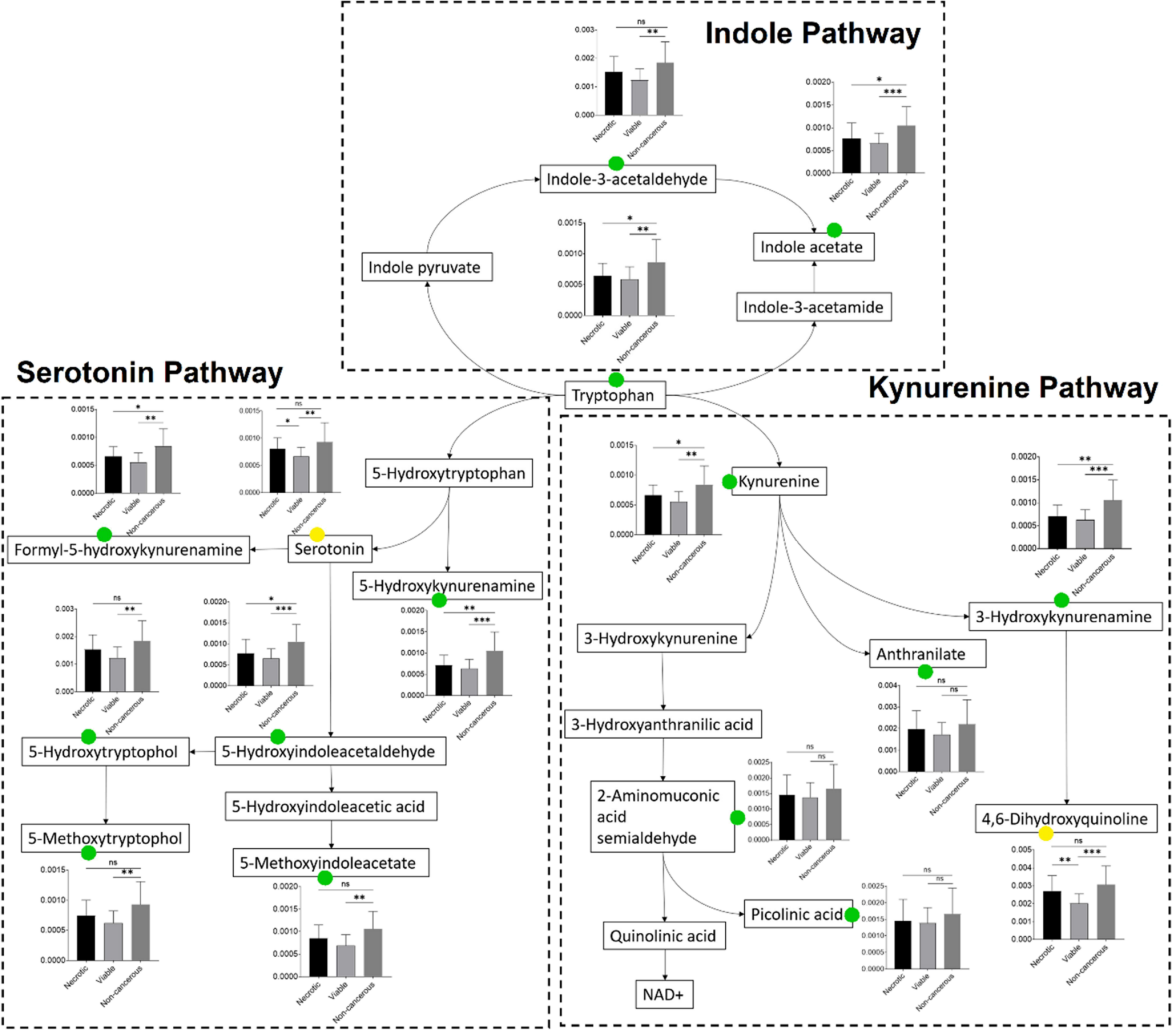
Metabolites found ubiquitous across GBM necrosis and viable tumor cells are mapped into a simplified tryptophan metabolic pathway. The bar graph shows the relative abundance of metabolites expressed as the ion intensity normalized to the TICs (black, necrotic; gray, viable; and dark gray, non-cancerous). The green circle indicates that the metabolite is ubiquitous across necrotic and viable cell regions. The yellow circle indicates metabolites (serotonin and 4,6-dihydroxyquinoline) with distinct relative abundances between the two regions. Most of the ubiquitous metabolites in viable tumor cells show a significantly altered relative abundance compared to the non-cancerous regions (*, *p* < 0.05; **, *p* < 0.01; ***, *p* < 0.001; ****, *p* < 0.0001; ns, not significant).

The majority of ubiquitous metabolites across necrotic and viable regions in all patients were mapped into tryptophan metabolism (Figure 5) and accounted for 19.66% of the total metabolites of this pathway. Most detected metabolites presented a relative abundance at similar levels between necrotic and viable cells, except for serotonin and 4,6-dihydroxyquinoline that were elevated in necrosis. Typically, over 95% of free tryptophan is degraded through the kynurenine pathway modulated by indoleamine-2,3-dioxygenase 1 (IDO1), IDO2, and tryptophan-2,3-dioxygenase.^56^ It was previously discovered that antitumor immune responses were inhibited in brain tumors through degradation of tryptophan by IDO1/2, which led to poor prognosis.^57^ This may explain the low relative abundance observed in tryptophan across both necrotic and viable regions compared to the non-cancerous regions. Kynurenine, an upstream metabolite in the kynurenine pathway, also showed a decreased abundance in this study, in agreement with a previous study that reported decreased tryptophan and kynurenine levels in blood from GBM patients compared to healthy individuals.^58^ Interestingly, differences in the relative abundance across these three histological regions were not observed in downstream metabolites such as 2-aminomuconic acid semialdehyde and picolinic acid. A number of ubiquitous metabolites were found related to the serotonin pathway of tryptophan metabolism. The overall relative abundance of metabolites was lower in the cancerous regions compared to the non-cancerous regions. It is noteworthy that the key metabolite, serotonin, was significantly reduced in the viable cells compared to both necrosis and non-cancerous zones. Apart from the well-established serotonin function as a neurotransmitter and its role in several psychiatric and neurological disorders, it has recently emerged as a growth factor in several types of tumor cells.^59^ Presented as a key feature of the GBM microenvironment, the effects of serotonin are highly dependent on the receptor subtypes expressed on tumor cells, which remain to be elucidated.^60^

Moreover, indole-3-acetaldehyde and indole acetate from the indole pathway were also found to be ubiquitous across necrosis and viable cells. This pathway was mediated by interleukin-4-induced-1 (IL4I1), and its expression was found inversely associated with overall survival in patients with gliomas.^61^

Other ubiquitous metabolites were found to be involved in arginine and proline metabolism, tyrosine metabolism, or histidine metabolism pathways (SI Figures S8–S10). However, due to the relatively small number of metabolites mapped and low pathway coverage, further investigation would be beneficial to confirm the role that these pathways play within the context of the tumor microenvironment.

### Structural Characterization of Important Metabolites with LESA-MS/MS

Acquiring structural information through MS/MS is an important way to confirm the putative annotation of an analyte.^62^ Despite the MS/MS capability, confirming the structure of metabolites using OrbiSIMS appeared to be challenging as the fragment ions of metabolites of interest could not be observed. Here, LESA-MS/MS was employed to extract metabolites from the same tissue samples analyzed by OrbiSIMS and generate MS/MS spectra for the confirmation of metabolite annotation (SI Figure S11). From the tryptophan metabolism pathway, the key metabolites including tryptophan, anthranilate, kynurenine, and picolinic acid were successfully identified based on their MS/MS spectra. Citrulline, proline, and ornithine from the proline and arginine metabolism, 3-methoxytyramine, tyrosine, and norepinephrine from the tyrosine metabolism, and histidine from the histidine metabolism were also identified by matching the fragmentation patterns (SI Figure S12). Some metabolites discovered in OrbiSIMS were not detected by LESA-MS/MS possibly due to deployment of different sampling and ionization mechanisms (SI annotations_identifications.xlsx).

## Conclusions

Overall, our proof-of-principle study using the novel application of OrbiSIMS has illustrated that this technique represents a new opportunity for untargeted metabolomics to probe intra-tumoral heterogeneity in GBM. First, we have shown that GBM cell subpopulations (necrotic, viable, and non-cancerous) within single tumors displayed distinct metabolite profiles. Therefore, it is possible that this analytical approach can be further developed to predict different histological features in GBMs, such as the clinically relevant infiltrative margin that harbors molecular signatures associated with residual disease. Second, by mapping ubiquitous metabolites across necrotic and viable regions into pathways, we discovered metabolic activities that were potentially essential for not only cancer cells with rapid proliferation (i.e., viable GBM cells) but also necrotic cells that often indicate poor prognosis and tumor recurrence. An important pathway discovered in this study was tryptophan metabolism, which has been reported in a number of GBM studies as a potential therapeutic target. In this study, we further elucidated its role in tumorigenesis within the tumor microenvironment.

This novel analytical strategy allowed in situ characterization of the tumor microenvironment and histopathological features with minimal sample preparation using a combined approach that offered high sensitivity, spatial resolution, and mass resolving power, which are difficult to achieve simultaneously with other analytical platforms. Also, this method is easily transferable to other types of tissue samples. Finally, metabolic characterization of the tumor tissue with cryogenic analysis in OrbiSIMS should be explored in future studies as it can potentially extend chemical coverage and increase the sensitivity of detection for certain biomolecule classes.

## Supporting Information Available

PCA of the ToF-SIMS image; comparison of data acquired with two modes of operation: Bi LMIG ToF-SIMS and Ar GCIB OrbiSIMS; histopathological regions shown on H&E-stained tissue sections; patient demographic information; multivariate analysis of depth profiling data; OrbiSIMS images; pathway analysis; metabolites mapped into arginine and proline, tyrosine, and histidine metabolism pathways; and LESA-MS/MS protocol and results (PDF)
annotations_identifications (XLSX)
