# Supplementary material for: Untargeted Metabolomic Characterization of Glioblastoma Intra-Tumor Heterogeneity Using OrbiSIMS
Source: Anal Chem. 2023 Mar 30;95(14):5994–6001. doi: 10.1021/acs.analchem.2c05807 (PMC10100400; doi:10.1021/acs.analchem.2c05807)
Supplement: Supplementary file 1 — ac2c05807_si_001.pdf [file ac2c05807_si_001.pdf]

# Supporting information for

# Untargeted Metabolomic Characterization of Glioblastoma Intra-Tumor Heterogeneity using OrbiSIMS

Wenshi He [†], Max K. Edney [‡], Simon ML Paine [§], Rian L. Griffiths [†], David J. Scurr [†], Ruman Rahman [‖], Dong-Hyun Kim [†]*

[†] Centre for Analytical Bioscience, Advanced Materials & Healthcare Technologies Division, School of Pharmacy, University of Nottingham, Nottingham, NG7 2RD, U.K.

[‡] Department of Chemical and Environmental Engineering, Faculty of Engineering, University of Nottingham, Nottingham, NG7 7RD, U.K.

[§] Neuropathology Laboratory, Nottingham University Hospitals NHS Trust, Nottingham, NG7 2UH, U.K.

[‖] Children's Brain Tumour Research Centre, Biodiscovery Institute, School of Medicine, University of Nottingham, Nottingham NG7 2RD, U.K.

* Email: dong-hyun.kim@nottingham.ac.uk

Supporting Information

Figure S1. PCA of ToF-SIMS image.

Figure S2. Comparison of data acquired with two modes of operation: Bi LMIG ToF-SIMS (lateral resolution ~ 1 μm) and Ar GCIB OrbiSIMS (lateral resolution 20 μm).

Figure S3. Depth profiling analysis of histopathological regions within single GBMs.

Figure S4. Microscopic images of H&E-stained tissue sections showing distinct histopathological regions within GBMs.

Table S1. Demographic information for patients included in the study.

Figure S5. PCA of depth profiling data (N = 4, n = 5).

Figure S6. Supervised model with OPLS-DA of depth profiling data.

Figure S7. The distribution of annotated discriminative ions from the depth profiling analysis visualized using Orbitrap imaging (pixel size 3 μm) of a tissue region containing a necrotic region and viable tumor cells.

Table S2. Essential metabolic pathways mapped from ubiquitous metabolites between necrotic and viable regions using MetExplore.

Figure S8. Metabolites mapped into the simplified arginine and proline metabolism pathway.

Figure S9. Metabolites mapped into the simplified tyrosine metabolism pathway.

Figure S10. Metabolites mapped into the simplified histidine metabolism pathway.

LESA-MS/MS protocol

Figure S11. LESA mechanism.

Figure S12. LESA-MS/MS for the identification of key metabolites discovered in OrbiSIMS.

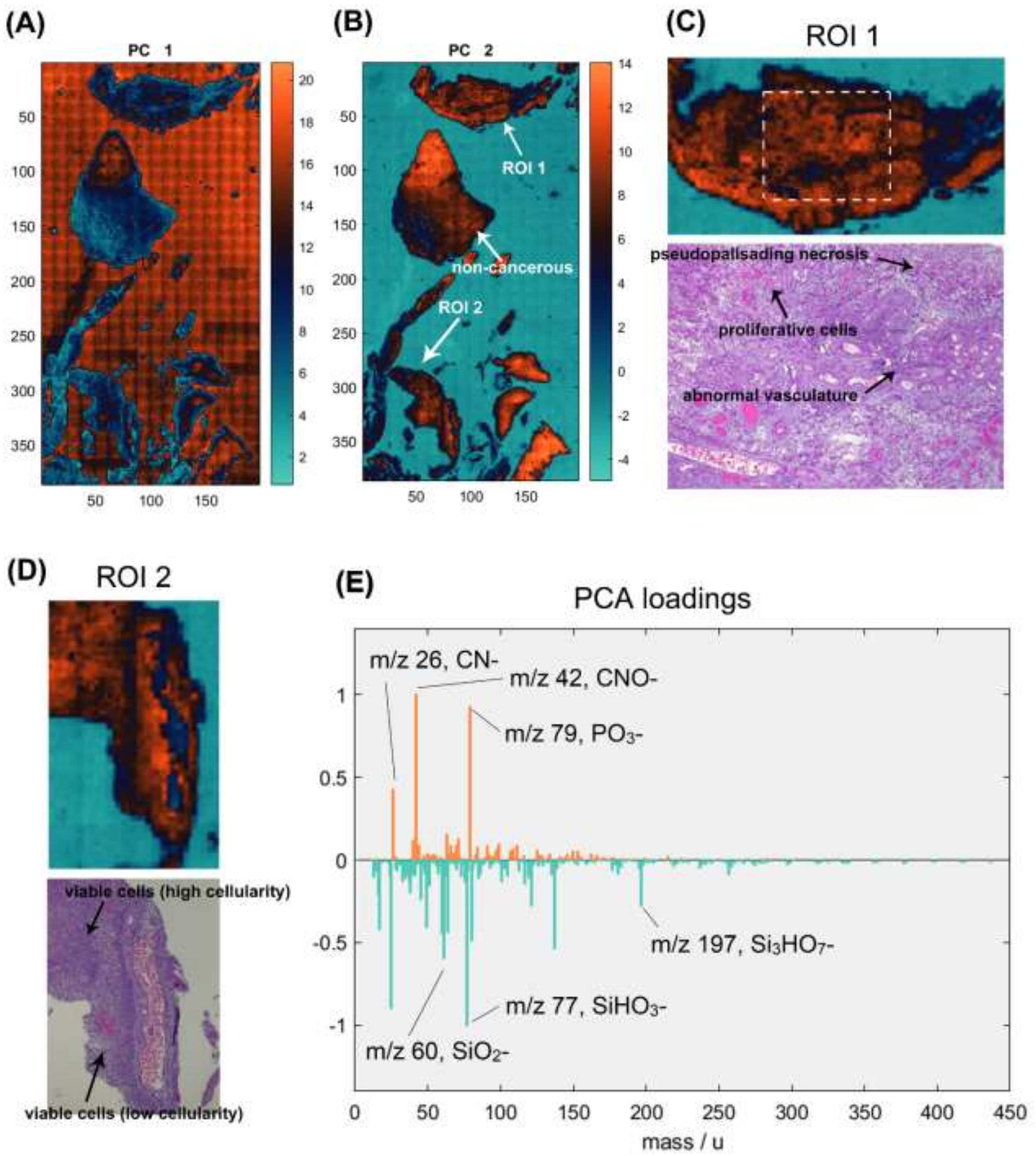


Figure S1. PCA of ToF-SIMS image. (A) PC 1 distinguishes the tissue fragments from background substrate (blue: tissue, red: substrate). (B) PC 2 visualizes the variance among different tissue regions. Regions of interest (ROIs) show high molecular heterogeneity and rich chemical information. (C) Visualization of PC 2 from GBM ROI 1 (red represent positive PC) and the H&E-stained tissue showing the histology of ROI 1. (D) Visualization of PC 2 from GBM ROI 2 (red represent positive PC) and the H&E-stained tissue showing the histology of ROI 2. (E) The PCA loadings plots show that the dominant ions in positive PC2 are small organic molecule fragments including CNO- (m/z 42), CN- (m/z 26), $PO_3$- (m/z 79), and dominant ions in negative PC2 are silicon substrate ions $SiO_2^-$ (m/z 60), $SiHO_3^-$ (m/z 77) and $Si_3HO_7^-$ (m/z 197).

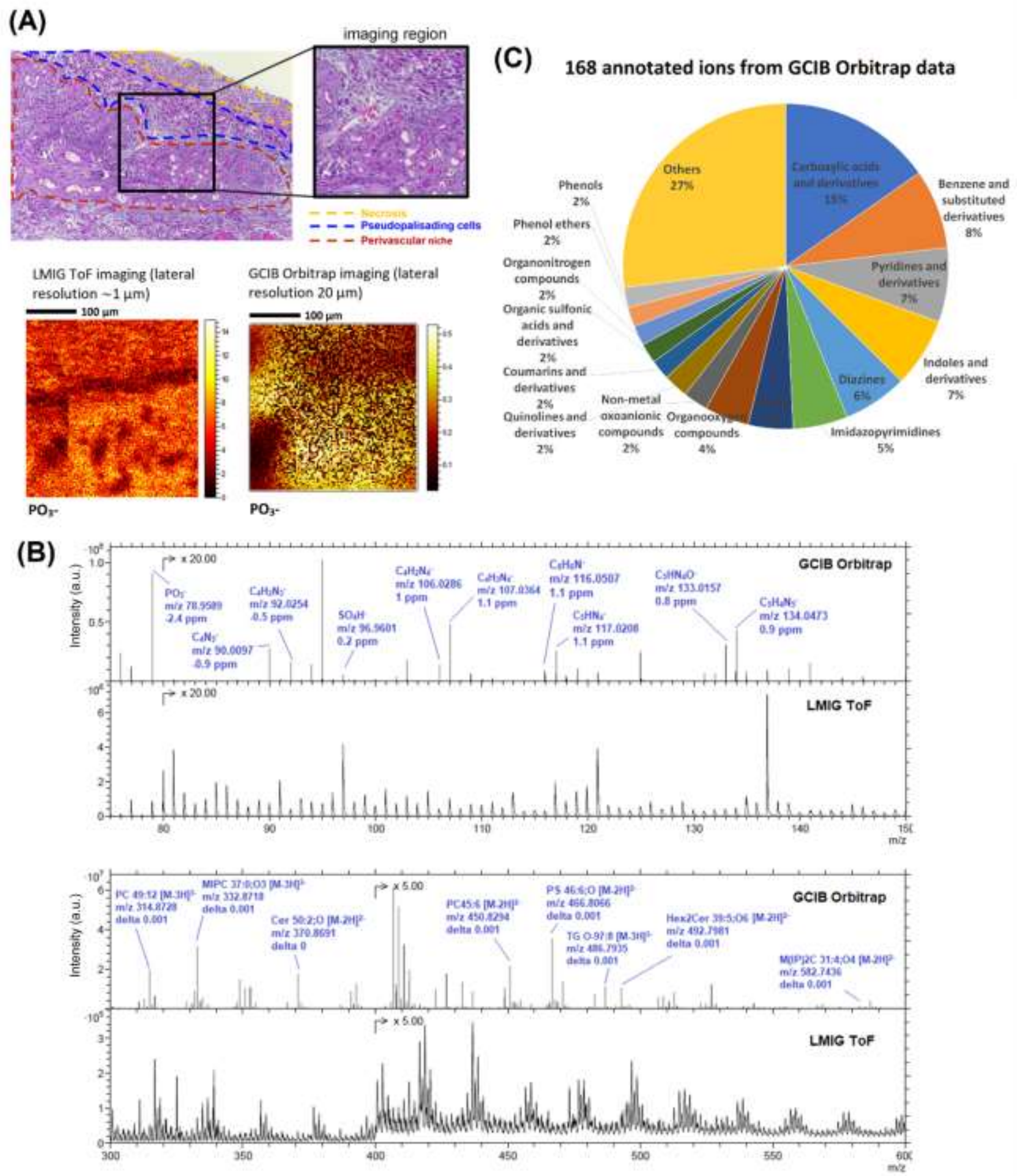


Figure S2. Comparison of data acquired with two modes of operation: Bi LMIG ToF-SIMS (lateral resolution ~ 1 µm) and Ar GCIB OrbiSIMS (lateral resolution 20 µm). (A) The distribution of phosphate ion ($PO_3^-$) from a region of interest on a morphologically heterogeneous tissue section visualized by ToF-SIMS and OrbiSIMS imaging. (B) The averaged mass spectra with peak annotations from the imaged area using GCIB Orbitrap and LMIG ToF methods. Higher number of small fragments (< m/z 150) are observed with LMIG ToF compared to GCIB Orbitrap, while higher mass accuracy and significantly improved detection of lipid species (m/z 300 – 600) are achieved by Ar GCIB OrbiSIMS imaging. (C) The chemical classes of 168 putatively annotated ions detected in negative mode as described in the Human Metabolome Database. Bi LMIG: bismuth liquid metal ion gun. Ar GCIB: argon gas cluster ion beam. ToF: time-of-flight. SIMS: secondary ion mass spectrometry.

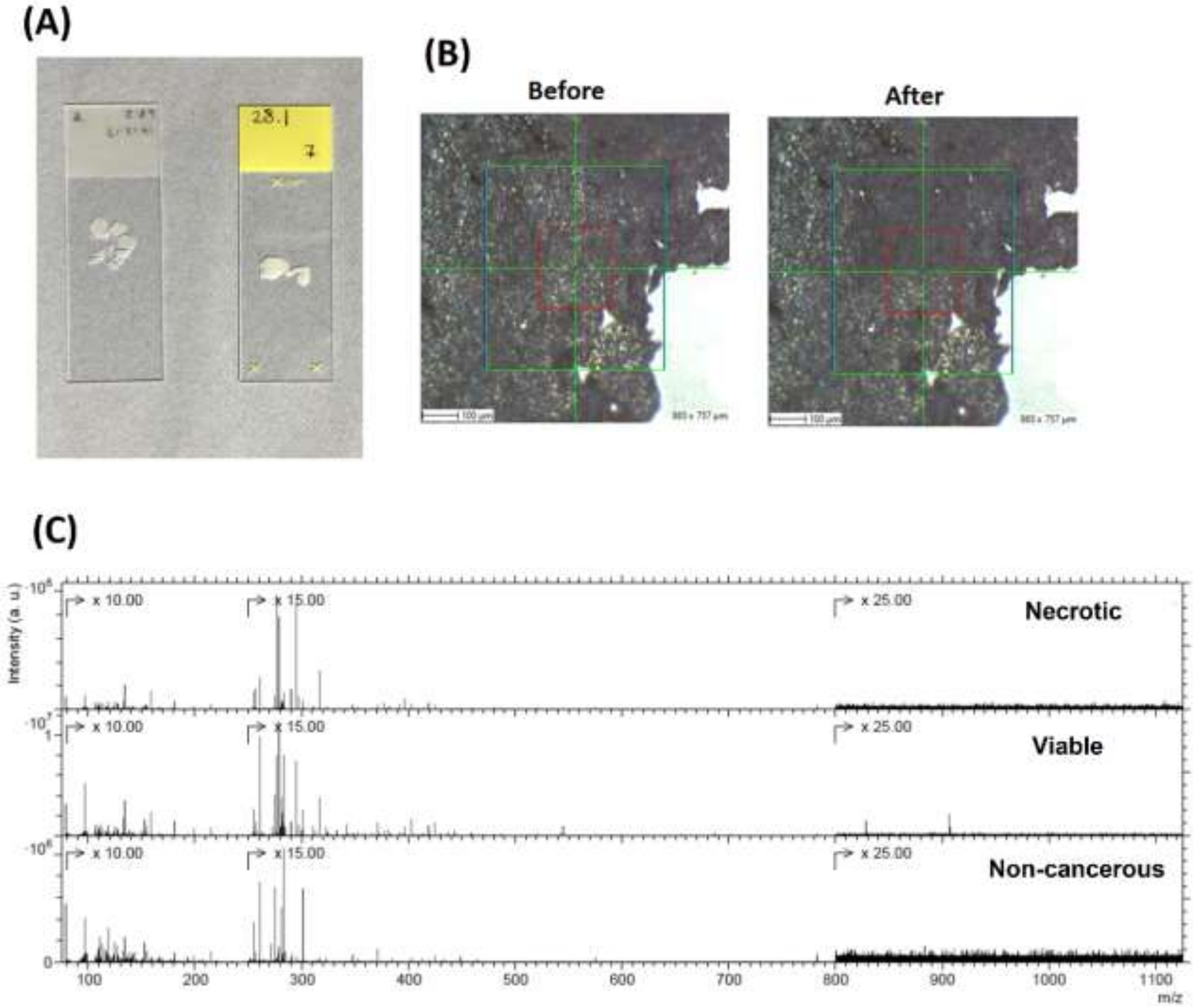


Figure S3. Depth profiling analysis of histopathological regions within single GBMs. (A) Example of GBM tissue sections after the paraffin removal with xylene. (B) Micro-view of a region of interest before and after depth profiling analysis with argon gas cluster ion beam using OrbiSIMS. (C) Representative spectra of non-cancerous, necrotic, and viable tumor cells regions from a GBM tissue section acquired by depth profiling using OrbiSIMS.

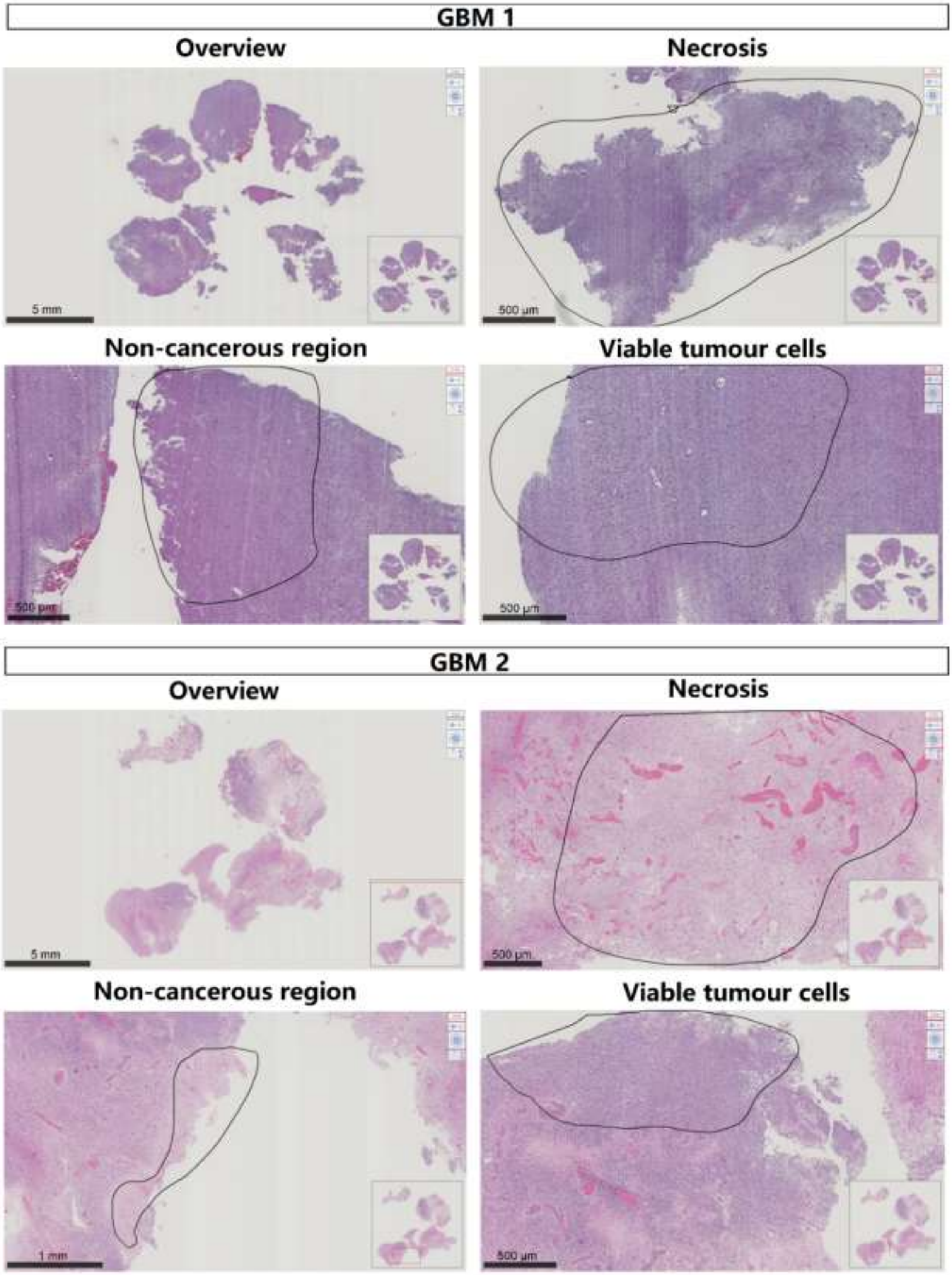


Figure S4. Microscopic images of H&E-stained tissue sections showing distinct histopathological regions within GBMs.

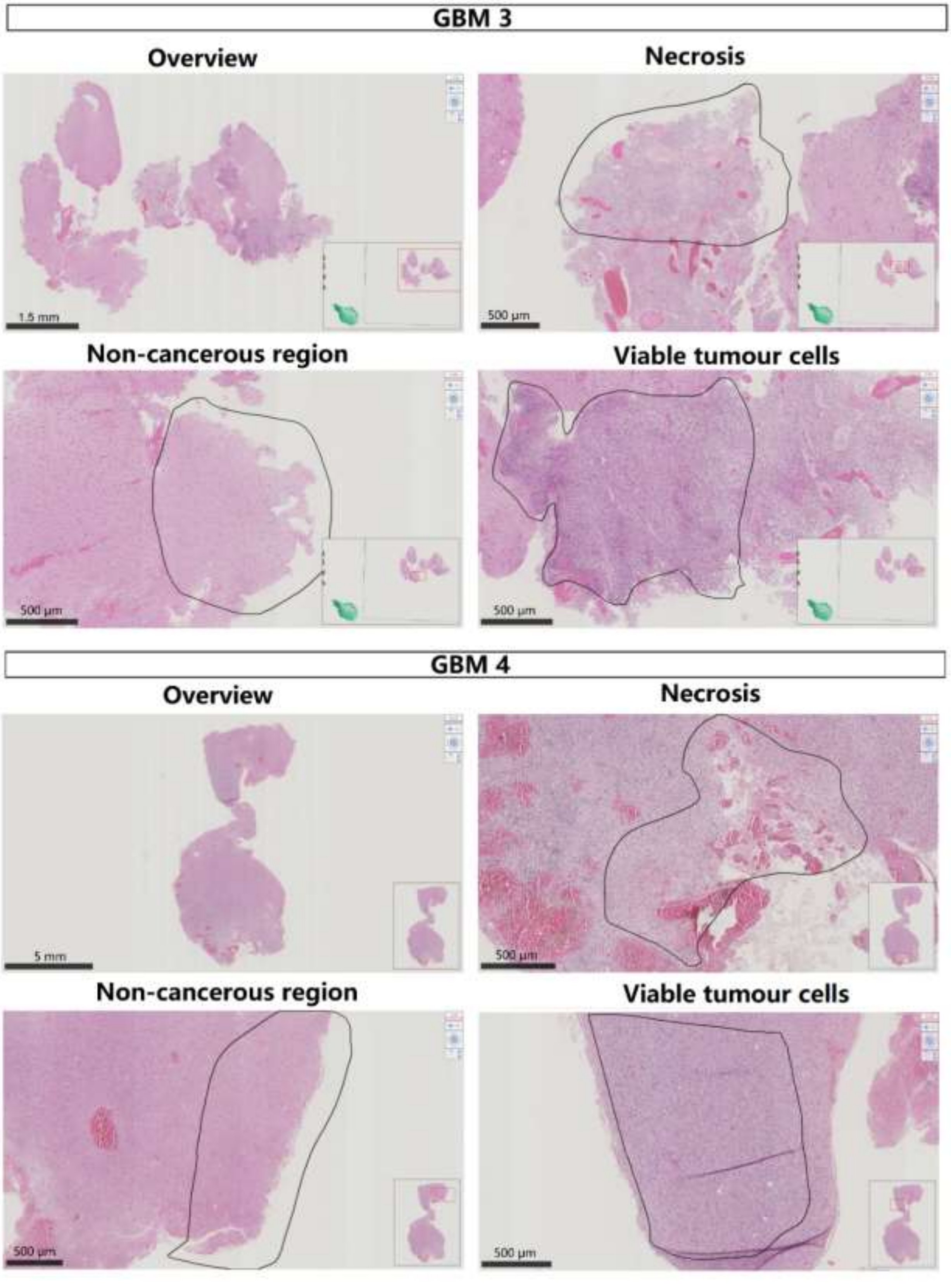


Figure S4. (continued) Microscopic images of H&E-stained tissue sections showing distinct histopathological regions within GBMs.

Table S1. Demographic information for patients included in the study.

| Samples | Regions | 5-ALA | Histology | IDH-1 | Primary tumor? | Tumor Site | Age | Sex | MGMT status |
|---|---|---|---|---|---|---|---|---|---|
| GBM 1 | Anterior enhancement | N | GBM | WT | Y | Right temporal | 66 | M | 0 |
| GBM 2 | Fluorescent core | N | MGNT | WT | Y | Left temporal | 39 | F | 25% |
| GBM 3 | Posterior margin | Y | GBM | WT | Y | Left temporal | 56 | F | 0 |
| GBM 4 | Superficial enhancement | Y | GBM | WT | Y | Right frontal | 71 | M | 0 |
| GBM - MS imaging only | Superficial enhancement | N | MGNT | WT | Y | Left temporal | 39 | F | 25% |
| GBM - Method development | Superficial rim | N | GBM | WT | Y | Left frontal | 68 | F | 0 |

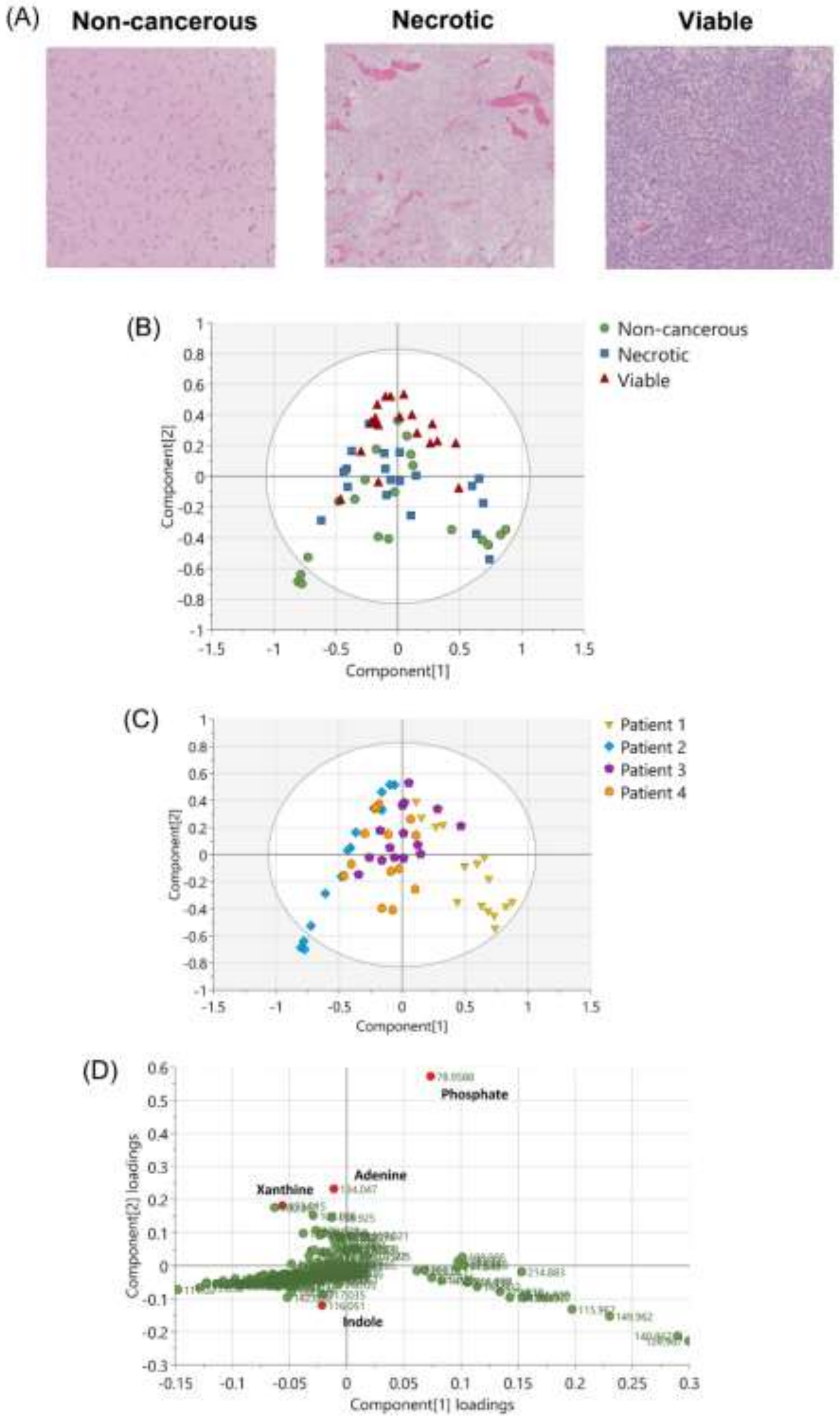


Figure S5. PCA of depth profiling data (N = 4, n = 5). (A) Representative microscopic images of morphologically distinct regions of GBM. (B) PCA plot grouped by morphological regions revealed the variations derived from the GBM microenvironment. (C) PCA plot grouped by patients showed inter-patient variations. (D) PCA loadings plot. In this unsupervised model, inter-patient variations were larger than the variations from tumor microenvironment because individual metabolome was inevitably affected by the presence of many irrelevant variables (i.e., intrinsic and extrinsic factors, such as age, gender, diet, lifestyle, and sample preparation), which is common in untargeted metabolomics. Therefore, supervised models were required to further identify microenvironment-related variable among the patients.

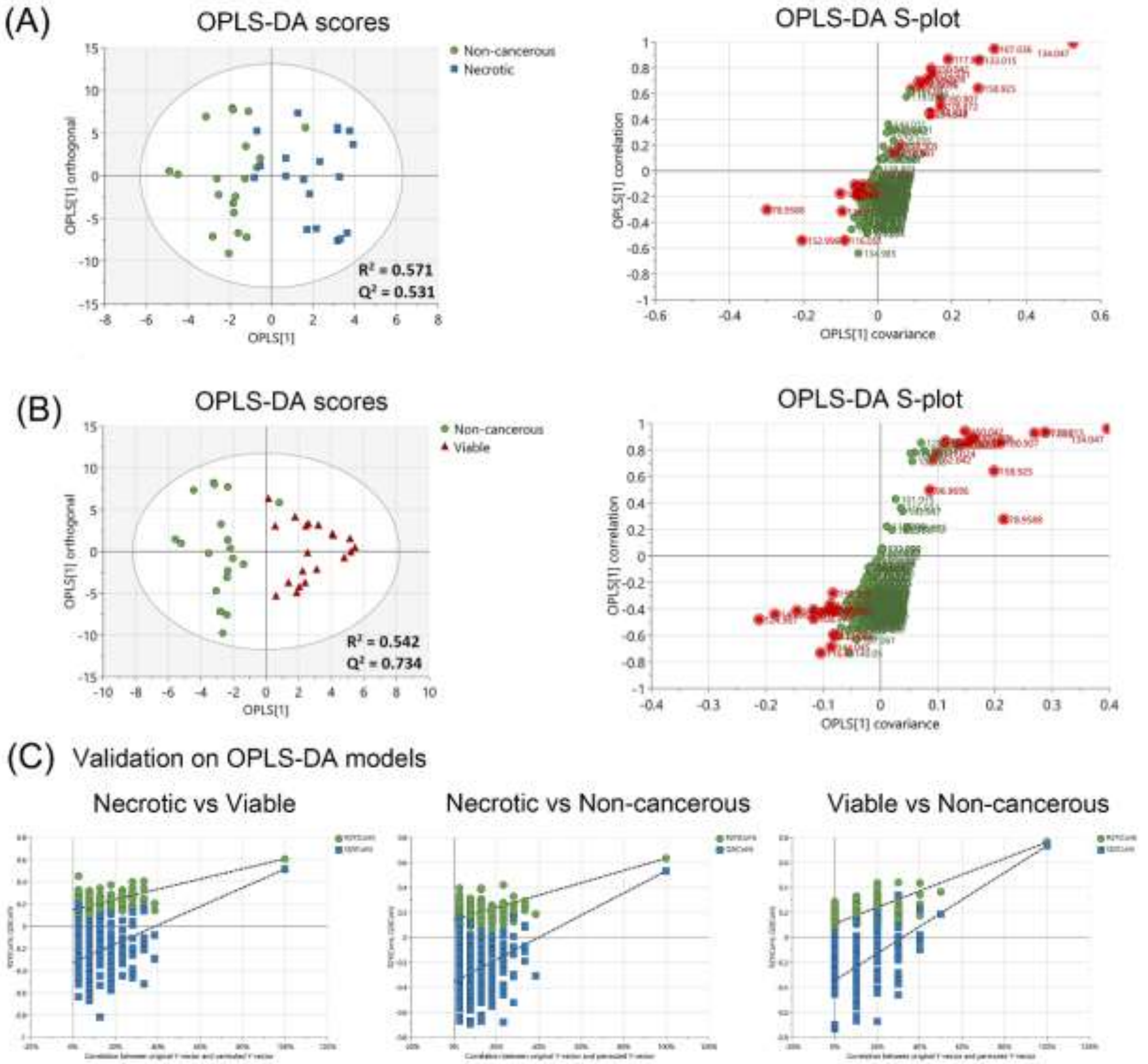


Figure S6. Supervised model with OPLS-DA of depth profiling data. (A) OPLS-DA scores plot and S-plot reveal differences in metabolite profiles between necrotic and non-cancerous regions ($R^2 = 0.571$, $Q^2 = 0.531$). (C) OPLS-DA score plot and the *S*-plot reveal differences in metabolite profiles between viable and non-cancerous regions ($R^2 = 0.542$, $Q^2 = 0.734$). (D) Permutation tests were performed on OPLS-DA models for statistical validation. The permutation plot based on 200 random permutations indicates that all models are valid as $Q^2$ values (blue squares) from the permuted test (bottom left) are lower than the corresponding original points (top right).

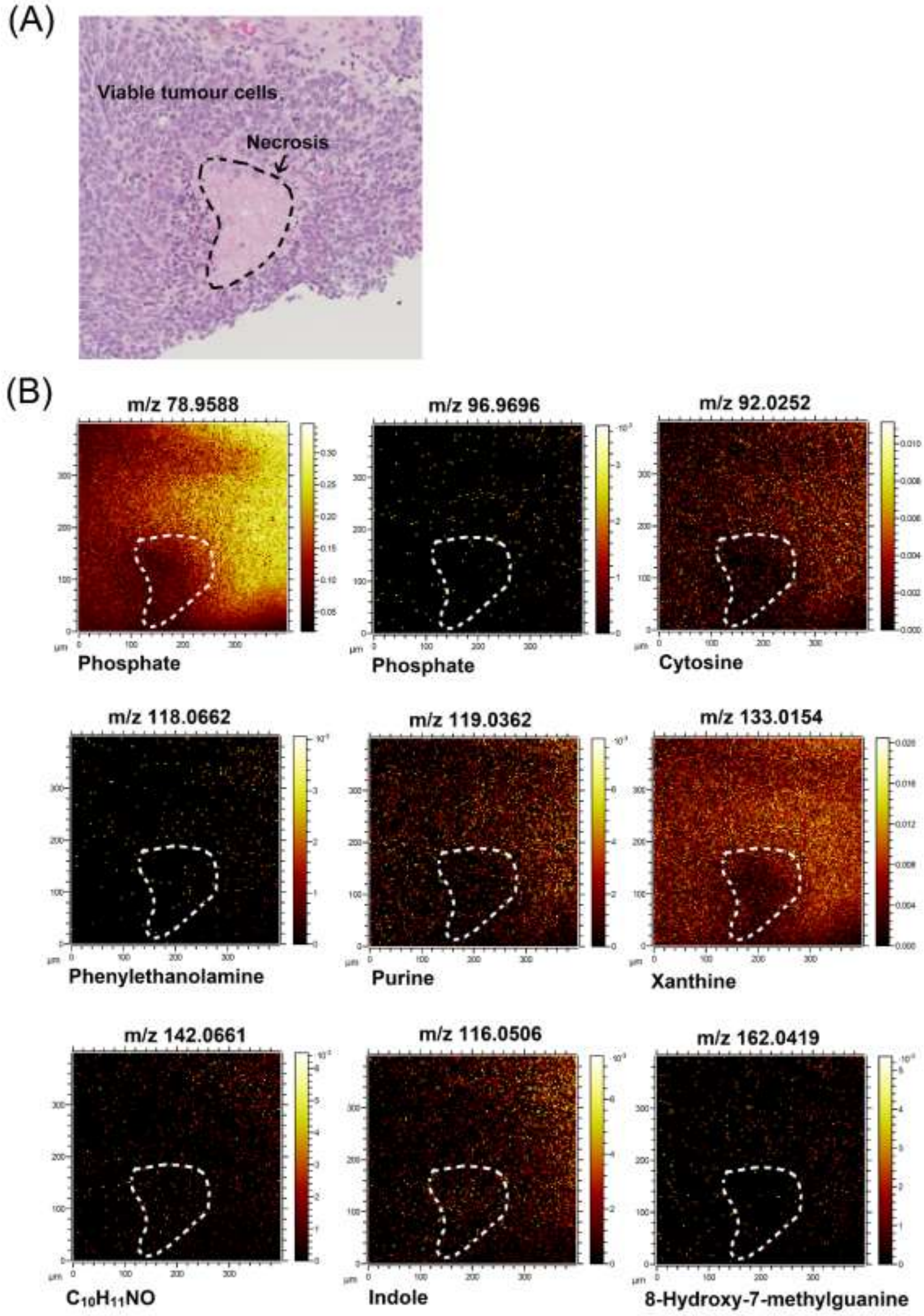


Figure S7. The distribution of annotated discriminative ions from the depth profiling analysis visualized using Orbitrap imaging (pixel size 3 μm) of a tissue region containing a necrotic region and viable tumor cells.

Table S2. Essential metabolic pathways mapped from ubiquitous metabolites between necrotic and viable regions using MetExplore. The over-representation of the mapped metabolites in each pathway is tested using a right tailed Fisher Exact Test. The *p*-values are corrected to account for the multiple tests performed for all pathways by Benjamini-Hochberg (BH) procedures.

| Name | Pathway coverage (%) | *p*-value | BH-corrected *p*-value |
|---|---|---|---|
| Tryptophan metabolism | 19.66 | $8.25\times10^{-14}$ | ***($4.54\times10^{-12}$) |
| Transport, extracellular | 5.53 | $4.44\times10^{-7}$ | **($9.49\times10^{-6}$) |
| Exchange/demand reaction | 5.8 | $5.17\times10^{-7}$ | **($9.49\times10^{-6}$) |
| Arginine and proline metabolism | 13.48 | $6.89\times10^{-6}$ | **$9.47\times10^{-5}$) |
| Tyrosine metabolism | 8.16 | $1.30\times10^{-4}$ | **($1.43\times10^{-3}$) |
| Histidine metabolism | 15.38 | $7.44\times10^{-4}$ | **($6.82\times10^{-3}$) |

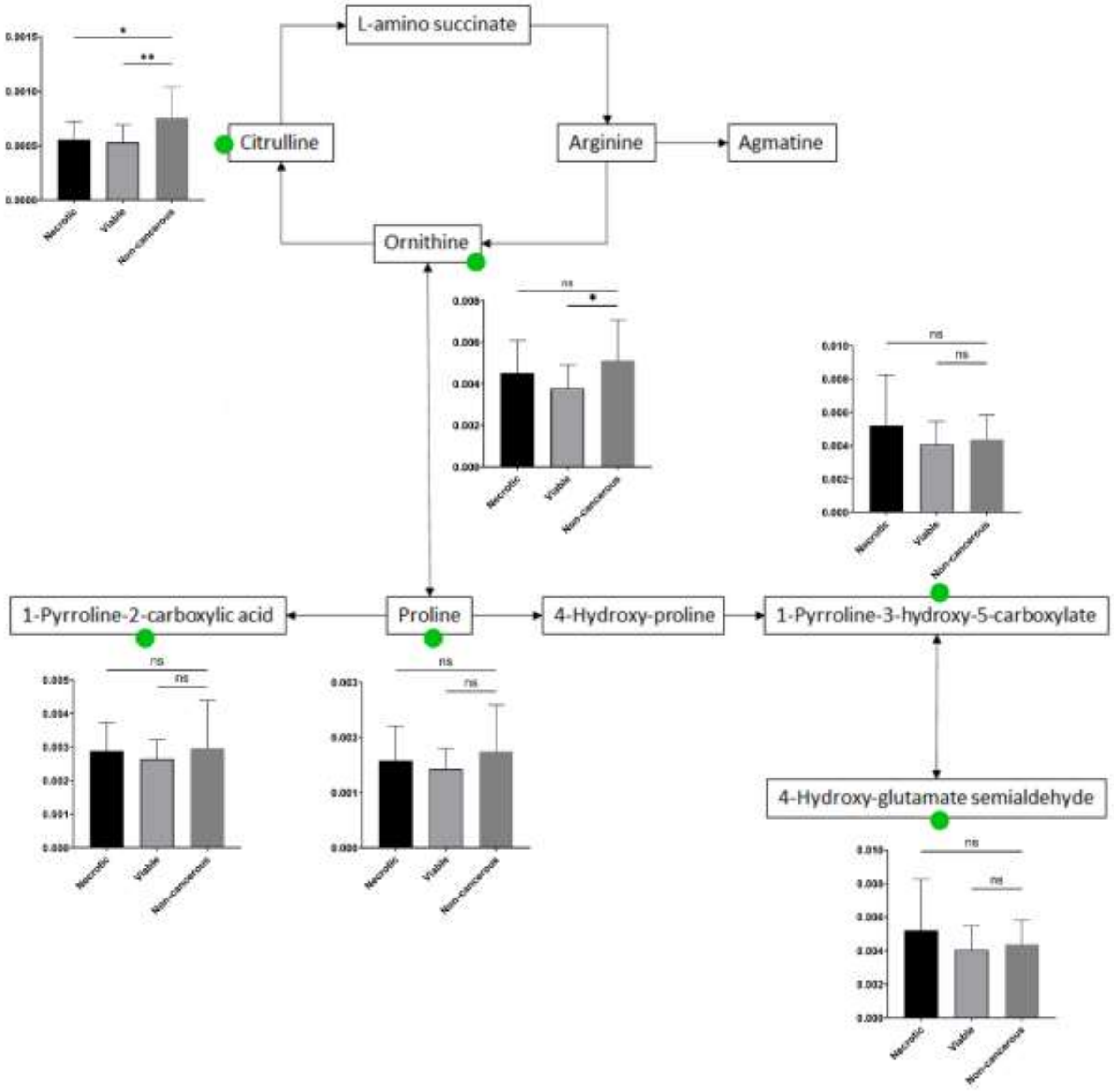


Figure S8. Metabolites mapped into the simplified arginine and proline metabolism pathway. The bar graph shows the relative abundance of metabolites expressed as ion intensity normalized to total ion counts. Green circle indicates that the metabolite is ubiquitous across necrotic and viable cell regions.

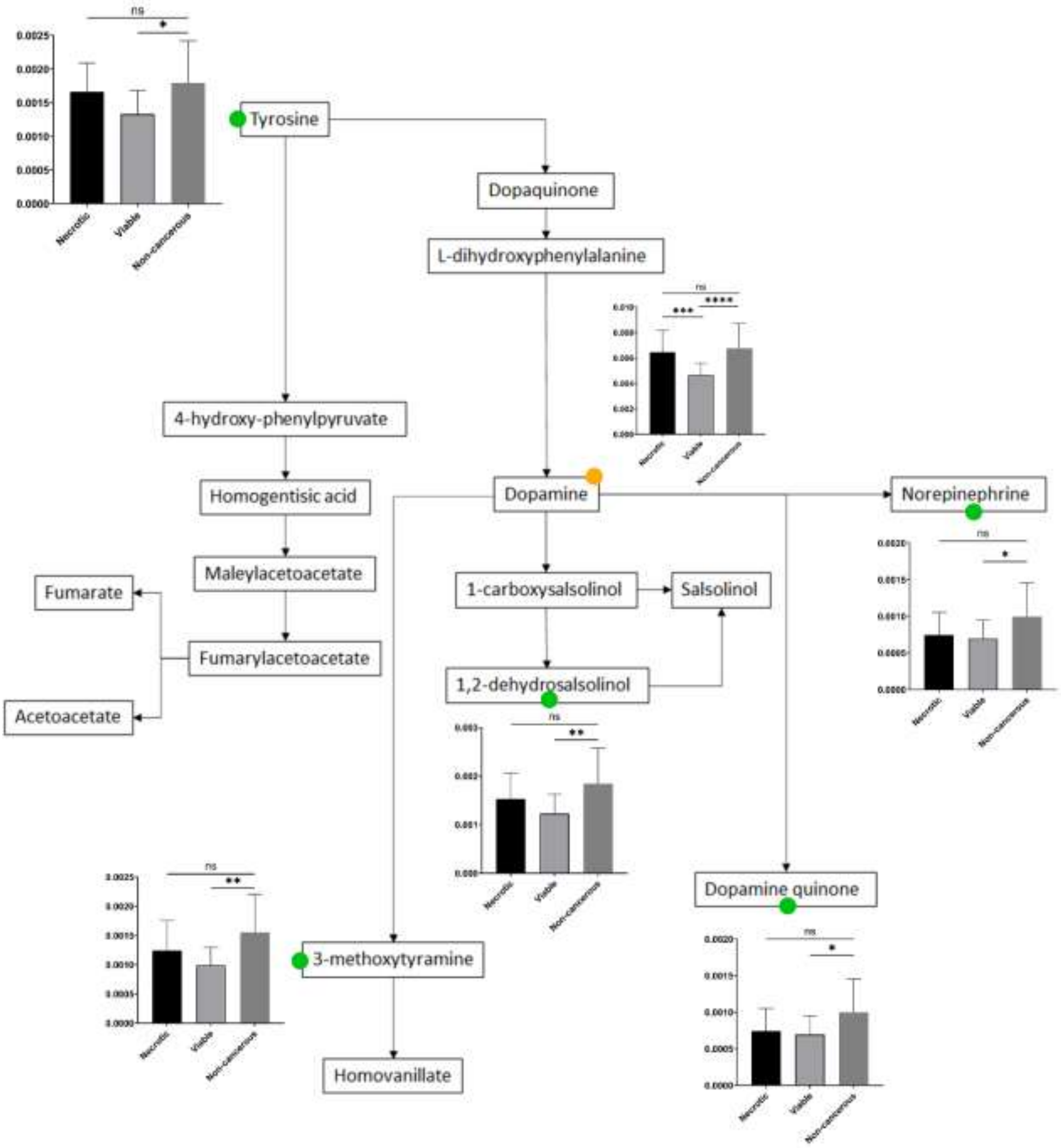


Figure S9. Metabolites mapped into the simplified tyrosine metabolism pathway. The bar graph shows the relative abundance of metabolites expressed as ion intensity normalized to total ion counts. Green circle indicates that the metabolite is ubiquitous across necrotic and viable cell regions. Yellow circle indicates metabolite (dopamine) with distinct relative abundances between the two regions.

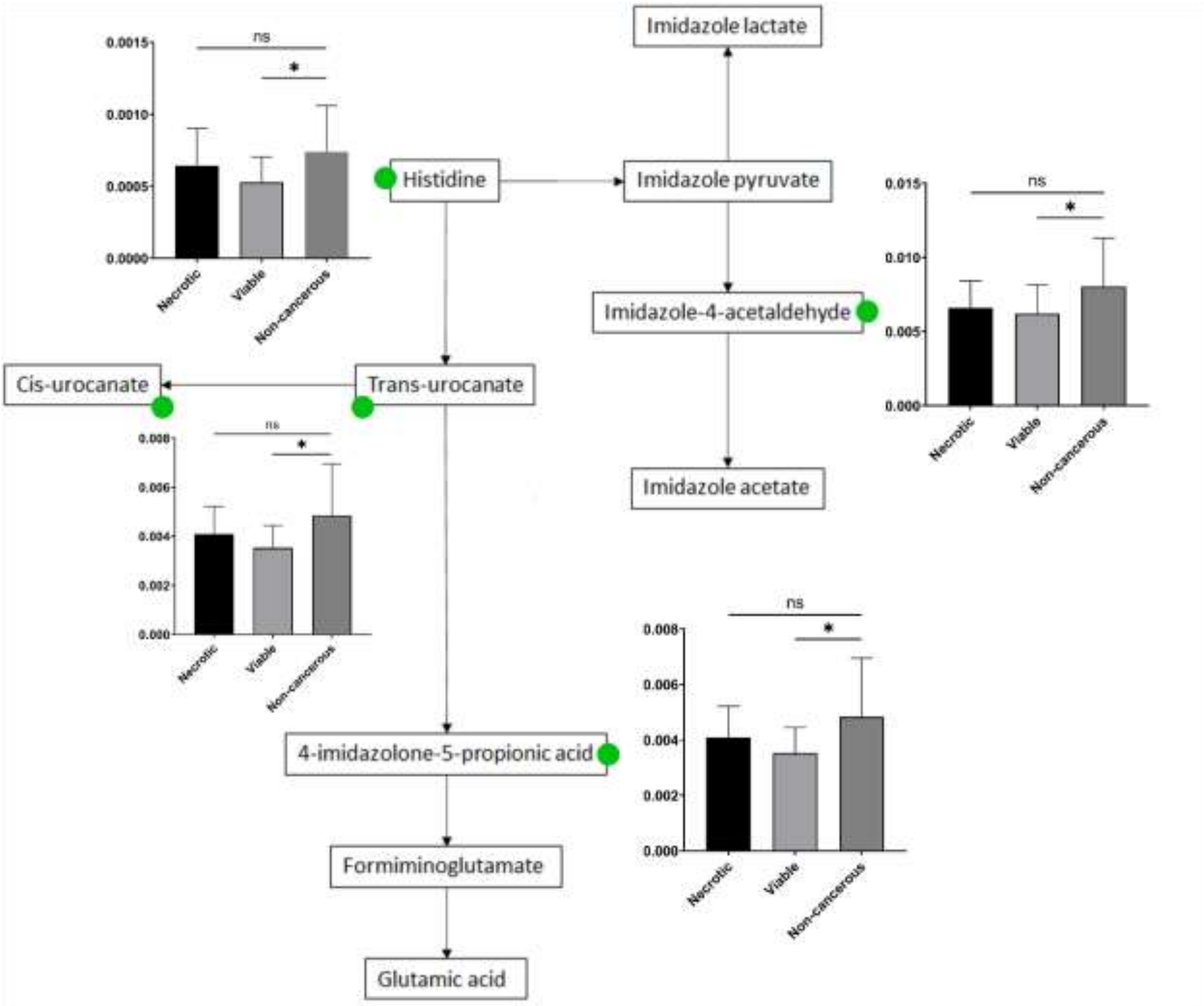


Figure S10. Metabolites mapped into the simplified histidine metabolism pathway. The bar graph shows the relative abundance of metabolites expressed as ion intensity normalized to total ion counts. Green circle indicates that the metabolite is ubiquitous across necrotic and viable cell regions.

LESA-MS/MS protocol

To acquire structural information for important metabolites, LESA-MS/MS experiments were carried out using a TriVersa Nanomate (Advion Biosciences, Ithaca, NY) coupled to a Q Exactive plus Orbitrap mass spectrometer (Thermo Scientific, San Jose, CA). Methanol and formic acid were purchased as MS grade (CHROMASOLV)from Sigma-Aldrich (Gillingham, UK). Deionized water was prepared using a Milli-Q water purification system (Millipore, MA, USA). First, 3.0 µL of an extraction solvent (methanol : water : formic acid 80:20:0.1) was aspirated from solvent reservoir with a pipette tip. Then, 1.5 µL extraction solvent was dispensed onto the GBM tissue surface and formed a stable liquid microjunction. After an incubation of 10 s, the extraction solvent containing extracted analytes was re-aspirated and delivered to the MS through electrospray ionization. The spray voltage was set to 1.5 kV with 0.5 psi gas pressure. Because it could not be determined prior to the experiment regarding which polarity in LESA-MS/MS allowed for the detection of the metabolites of interest, the experiment was operated in both positive and negative mode for a total of 2 min using a mass scan range of m/z 70–500 and an inclusion list. The mass resolution was set to 140,000 at m/z 200 for full MS scans and 35,000 at m/z 200 for MS/MS. For full MS, maximum injection was 200 ms with AGC target $3 \times 10^6$. For MS/MS acquisition, the AGC target was set to $2 \times 10^5$ and the stepped collision energy of 15, 30 and 45 eV were applied.

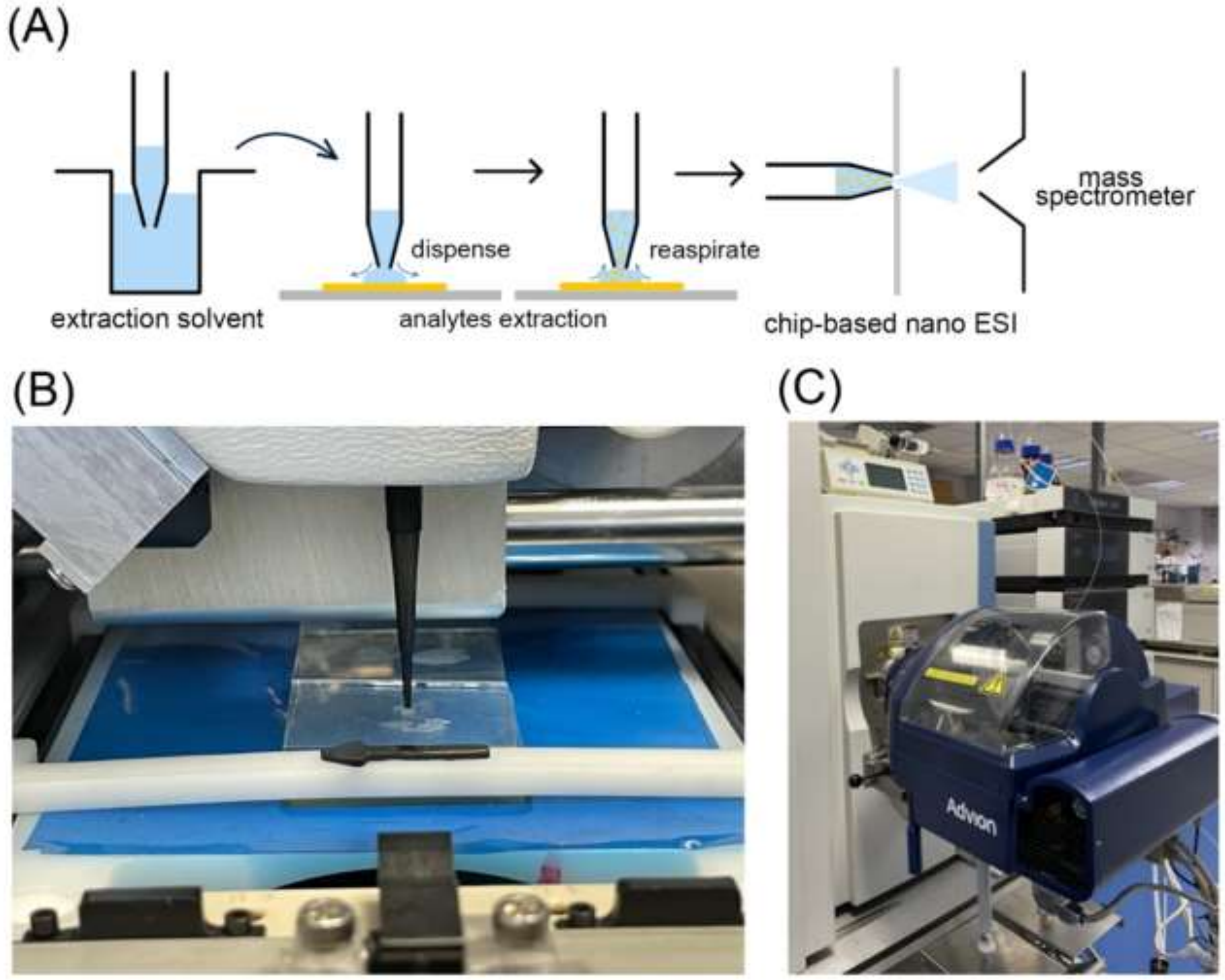


Figure S11. LESA mechanism. (A) LESA sampling schematic workflow. (B) a liquid microjunction is formed between the pipette tip and the GBM tissue section. (C) Advion NanoMate chip-based infusion nanoESI system coupled to a high-resolution Orbitrap mass spectrometer

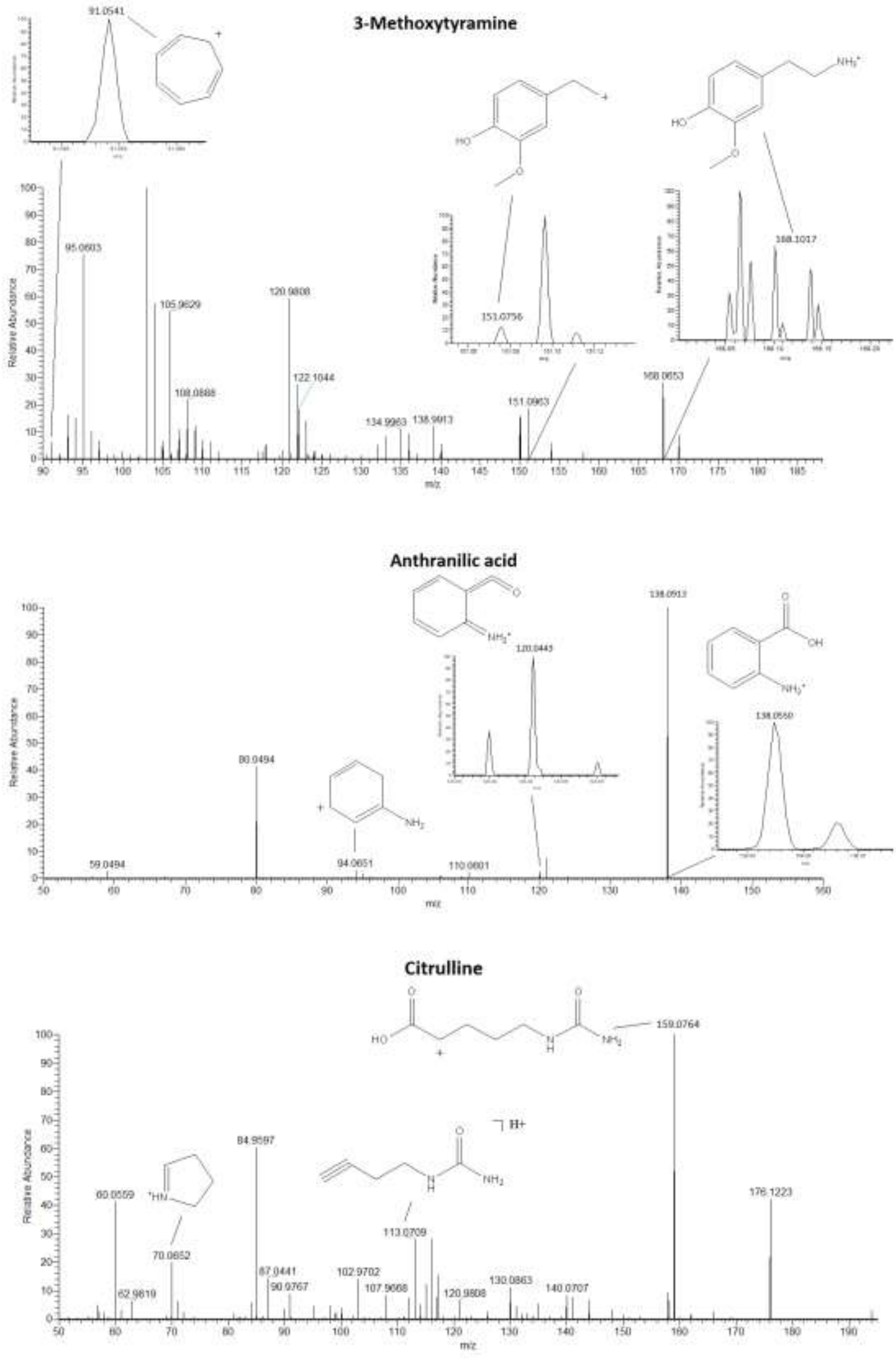


Figure S12. LESA-MS/MS for the identification of key metabolites discovered in OrbiSIMS. The mzCloud database was used for pattern matching and annotation of fragment ions.

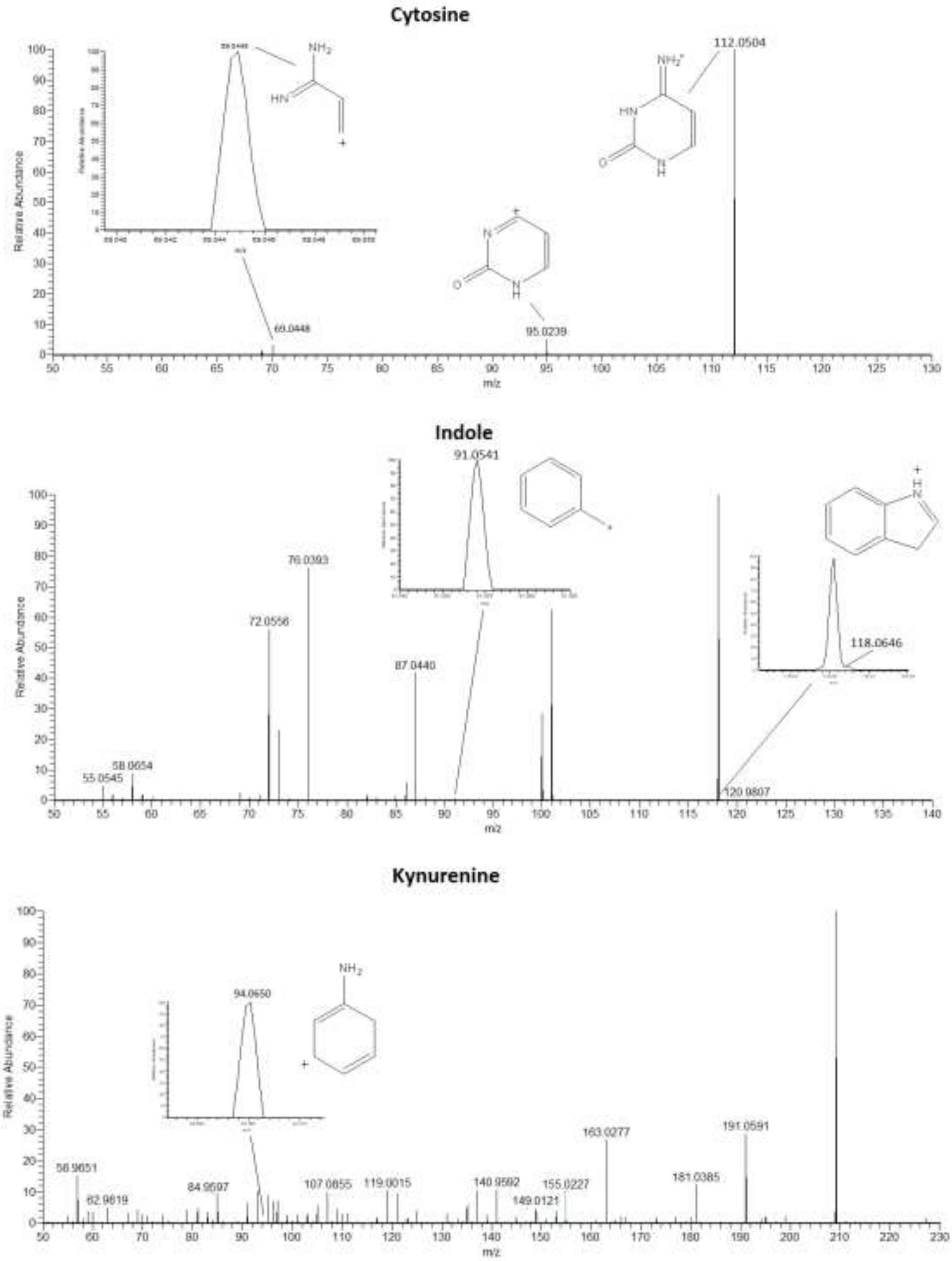


Figure S12. (continued) LESA-MS/MS for the identification of key metabolites discovered in OrbiSIMS. The mzCloud database was used for pattern matching and annotation of fragment ions.

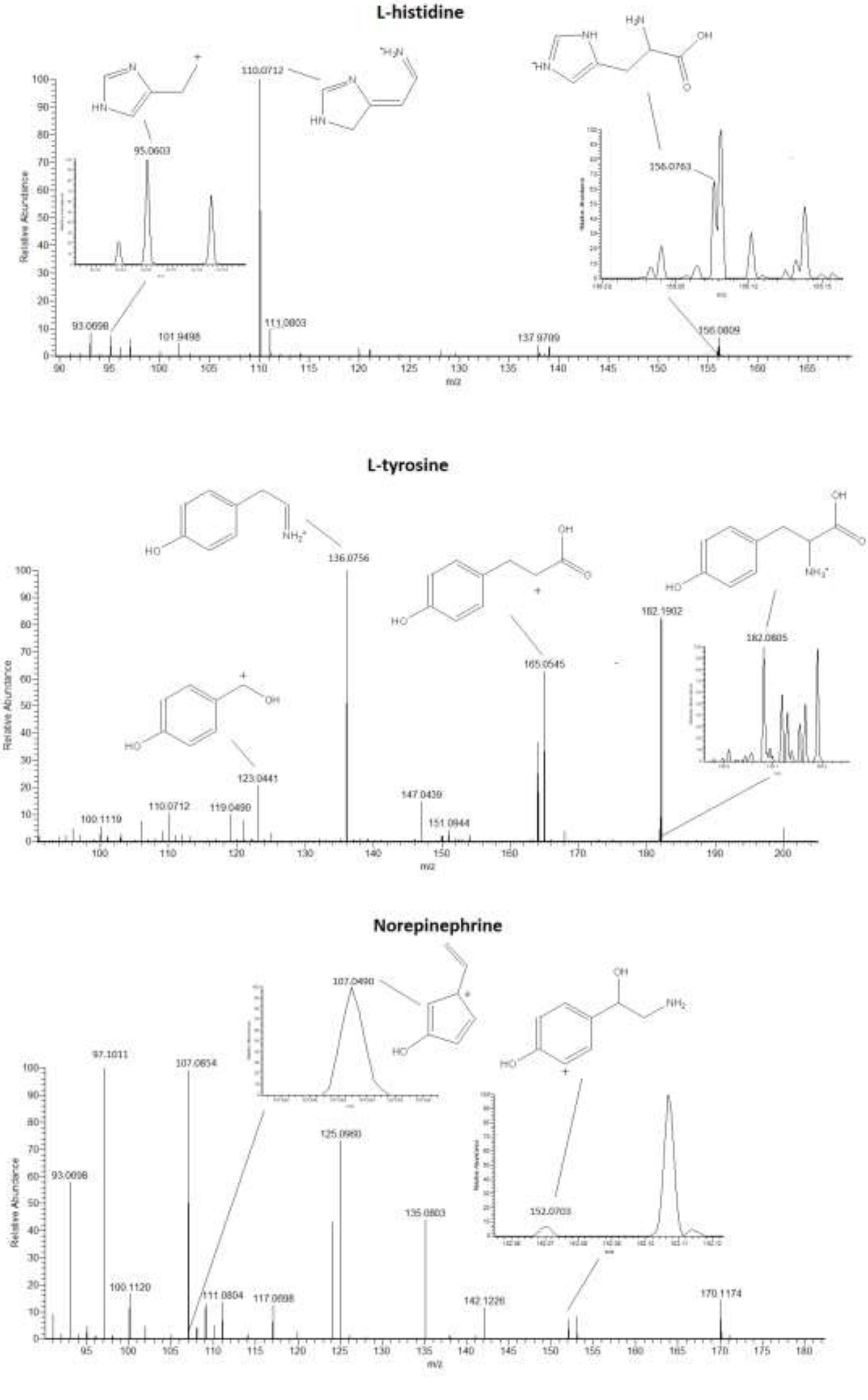


Figure S12. (continued) LESA-MS/MS for the identification of key metabolites discovered in OrbiSIMS. The mzCloud database was used for pattern matching and annotation of fragment ions.

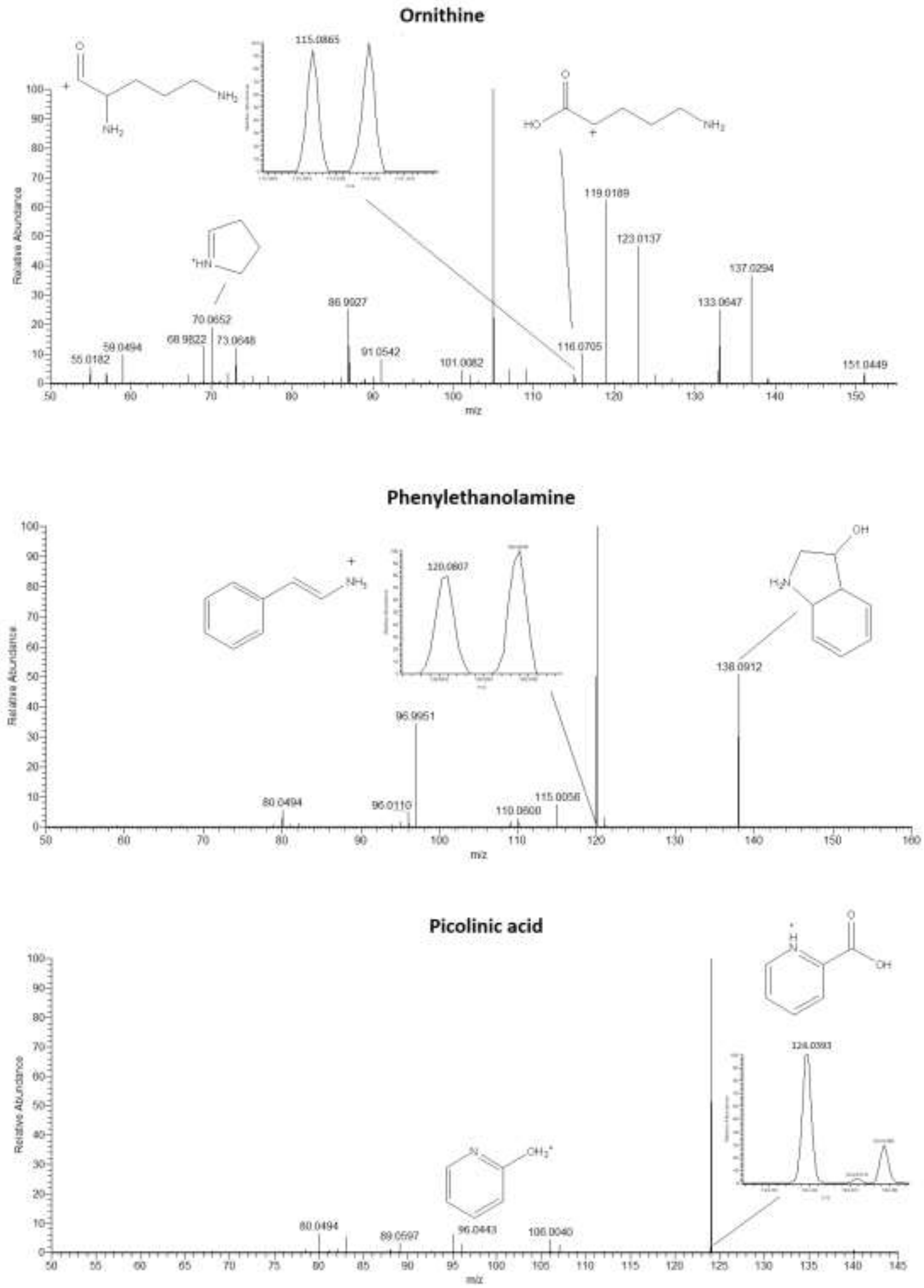


Figure S12. (continued) LESA-MS/MS for the identification of key metabolites discovered in OrbiSIMS. The mzCloud database was used for pattern matching and annotation of fragment ions.

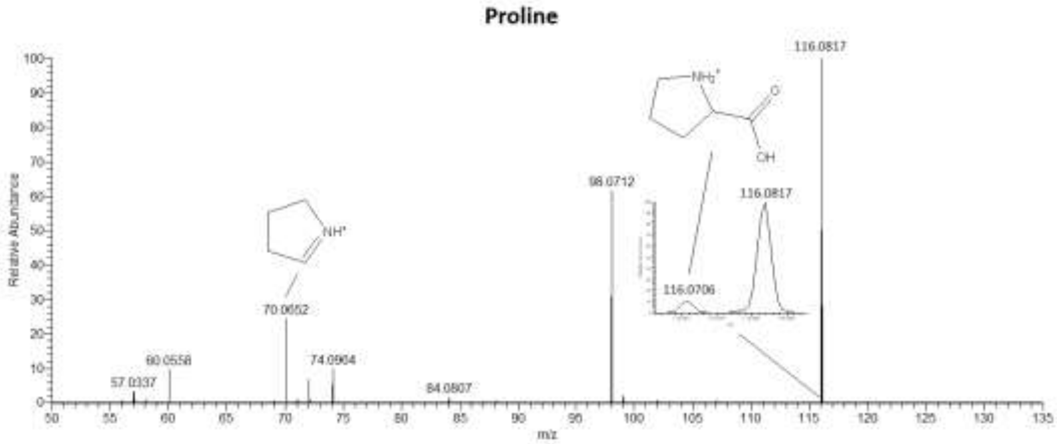


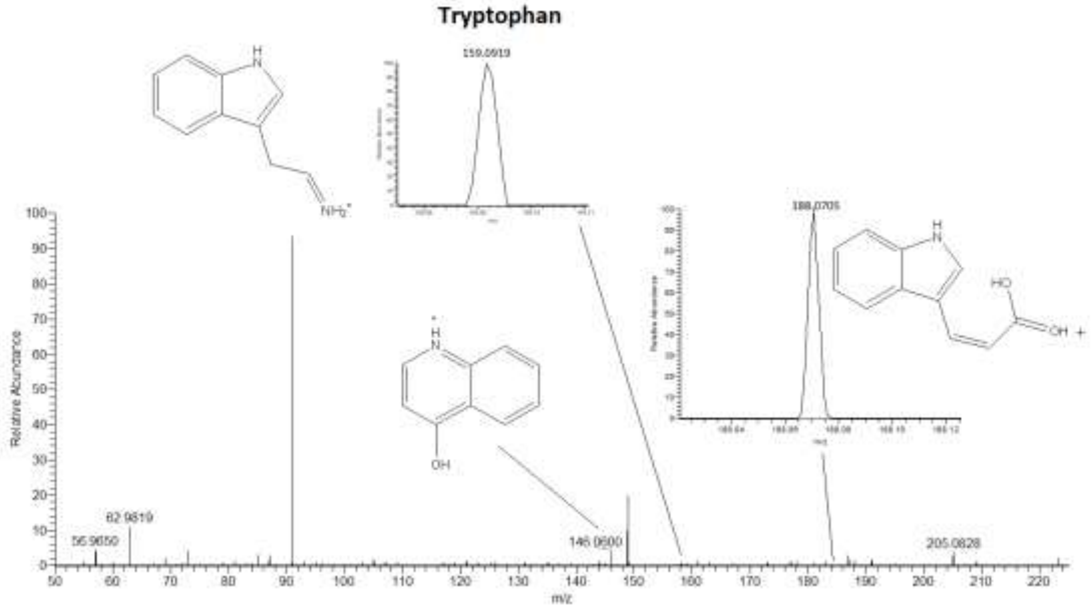


Figure S12. (continued) LESA-MS/MS for the identification of key metabolites discovered in OrbiSIMS. The mzCloud database was used for pattern matching and annotation of fragment ions.
